# SUMOylation inhibition potentiates the glucocorticoid receptor to program growth arrest of acute lymphoblastic leukemia cells

**DOI:** 10.1038/s41388-025-03305-3

**Published:** 2025-02-14

**Authors:** Emma Valima, Vera Varis, Kseniia Bureiko, Joanna K. Lempiäinen, Anna-Mari Schroderus, Laura Oksa, Olli Lohi, Tuure Kinnunen, Markku Varjosalo, Einari A. Niskanen, Ville Paakinaho, Jorma J. Palvimo

**Affiliations:** 1 Institute of Biomedicine, University of Eastern Finland, Kuopio, Finland; 2 Institute of Clinical Medicine, University of Eastern Finland, Kuopio, Finland; 3 Tampere Center for Child, Adolescent, and Maternal Health Research, Tampere University, Tampere, Finland; 4 Tampere University Hospital, Tays Cancer Center, Tampere, Finland; 5 ISLAB Laboratory Centre, Kuopio, Finland; 6 Institute of Biotechnology, HiLIFE, University of Helsinki, Helsinki, Finland; 7 HiLIFE-Proteomics Unit, University of Helsinki, Helsinki, Finland

**Keywords:** Acute lymphocytic leukaemia, Sumoylation

## Abstract

Glucocorticoids are a mainstay in the treatment of B-cell acute lymphoblastic leukemia (B-ALL). The glucocorticoid receptor (GR), a ligand-activated transcription factor (TF), mediates their actions. Chromatin occupancy, chromatin-protein networks (chromatomes) and gene programmes of GR are regulated by SUMOylation, a post-translational modification with therapeutic implications in other hematomalignancies. To unravel the GR-SUMOylation crosstalk in B-ALL, we induced hypoSUMOylation in NALM6 B-ALL cells with a SUMOylation inhibitor (SUMOi, ML-792). Genome-wide profiling of GR and SUMO chromatin-binding and chromatin accessibility revealed that hypoSUMOylation augmented GR chromatin occupancy and altered chromatin openness. Association with transcriptome data indicated that the hypoSUMOylation-induced GR-binding sites predominantly repressed genes associated with cell cycle and DNA replication. Consistently, hypoSUMOylation potentiated glucocorticoid-induced cell cycle arrest and growth suppression. Moreover, our proteomic analyses revealed that the protein network of chromatin-bound GR is tightly intertwined with SUMO2/3 and that SUMOylation modulates the stability of the network. The chromatome contained several B-cell TFs with cognate binding motifs found on GR-adjacent chromatin sites, indicating their simultaneous occupancy on chromatin. In sum, our data imply potential for targeting SUMOylation to increase sensitivity to glucocorticoids in B-ALL, supported by ex vivo data of glucocorticoid and SUMOi TAK-981 combination-treated B-ALL patient samples.

## Introduction

Highly aggressive acute lymphoblastic leukemia (ALL) is the most prevalent pediatric cancer, accounting for approximately one third of all childhood cancer diagnoses [1]. Majority of ALL cases comprise of B-cell acute lymphoblastic leukemia (B-ALL), a subtype affecting precursors of B-lymphocyte lineage. Despite remarkable advancements in the treatment of B-ALL in the last few decades, the therapeutic regimen is in many cases still suboptimal. Approximately 15% of adult patients are resistant to initial therapy, and relapses are relatively common, affecting 15–20% of patients [2, 3]. Strikingly, the prognosis of patients with recurrent disease remains poor [3].

Glucocorticoids (GCs), such as dexamethasone (Dex), are a mainstay in the frontline combination chemotherapy against ALL [4, 5]. The essential contribution of GCs to successful therapy is highlighted by the fact that the overall treatment response correlates with response to GCs alone [6]. Yet, GCs demonstrate several issues, such as varying degrees of efficacy, development of GC resistance or unresponsiveness. The prognosis for patients suffering from these issues remains poor [4, 5, 7]. Prolonged exposure to high-dose GCs during treatment also poses a risk for side effects, such as myopathies, bone morbidities and increased susceptibility to severe infections [4]. The therapeutic effects of GCs are mediated by the glucocorticoid receptor (GR), a nuclear receptor that directly regulates gene expression by acting as a ligand-activated transcription factor (TF). The therapeutic effects of GCs are usually attributed to their ability to induce cell death in the leukemic cells; although the exact mechanisms by which GCs exert their cytotoxic effects have remained elusive, GR-mediated regulation of target genes has been shown to be essential [8, 9].

SUMOylation, covalent conjugation of small ubiquitin-like modifier (SUMO) proteins, has emerged as a potential target in the treatment of various cancers, including other hematomalignancies [10–14]. A vast array of proteins, in particular those in the nucleus, including various TFs, are targeted and regulated by SUMOylation [15, 16]. Importantly, SUMOylation regulates the activity and chromatin occupancy of GR, as well as the composition of its chromatin-protein network [17, 18]. The SUMO family contains multiple isoforms, of which SUMO1, -2 and -3 are the best studied. As SUMO2 and SUMO3 are virtually (97%) identical and thus largely indistinguishable experimentally, they are from now on collectively referred to as SUMO2/3 [19, 20]. Notably, cancer cells generally exhibit higher levels of SUMO2/3 than SUMO1 [21]. SUMOylation of target proteins occurs via a highly dynamic cascade consisting of the dimeric SUMO-activating enzyme (SAE1/2), the SUMO-conjugating enzyme (UBC9) and a set of assisting SUMO ligases, such as PIAS1 [15, 22]. Conjugated SUMOs are cleaved from target proteins and released for a new cycle of SUMOylation by SUMO-specific proteases (SENPs) [23].

Targeting SUMOylation has shown promise as a therapeutic approach in acute myeloid leukemia (AML). In acute promyelocytic leukemia, a subtype of AML characterized by the oncogenic fusion receptor PML-RARα, triggering SUMOylation of the receptor restores signaling through its normal non-fusion counterpart [11]. In other AMLs, inhibition of SUMOylation has been indicated to help overcome chemoresistance and improve the efficiency of differentiation therapies [12–14], and more recently suppress AML growth independently of immunity reactions [24]. Mild inhibition of the modification has also been reported to facilitate differentiation of leukemic promyelocytes into granulocytes in a cell model system [25]. Interestingly, SUMOylation pathway components are often dysregulated in cancers, with a bias towards elevated levels [10]. For example, SAE1, SAE2, UBC9 and SUMO2/3 are overexpressed in high MYC-expressing B-cell lymphomas and elevated SUMOylation signature is associated with higher AML risk and poorer survival [10, 26]. In multiple myeloma (MM), hyperactivity of the SUMOylation pathway has been linked to adverse patient outcomes [27]. In B-ALL, SUMOylation pathway components have been identified among resistance-relapse genes based on misexpression (relapse vs. diagnosis) and the effect of B-ALL cell growth [28]. Moreover, genes of the pathway components have recently been identified as rare cancer driver genes in this disease [29]. Yet, despite the accumulating evidence of the pathway’s significance in other hematological disorders, the role of SUMOylation in B-ALL context has remained unexplored.

Here, to gain more insight into how SUMOylation influences GR action in B-ALL, we used a multi-omics approach to explore the crosstalk between SUMOylation and GC signaling on a genome- and proteome-wide level. We used a potent inhibitor of SAE, ML-792 [30] (SUMOi), to induce a hypoSUMOylated state in NALM6 cells, a B-ALL derived human pre-B cell line, which has been used as an in vitro disease model and is sensitive to Dex [28, 31–33]. In addition to examining the chromatin binding patterns of GR and SUMO2/3 and changes in gene expression with ChIP-seq and RNA-seq, we assessed changes in chromatin openness with ATAC-seq. Using chromatin-directed proteomics, we identified several shared TFs and coregulators associated with chromatin-bound GR and SUMO2/3, including B-cell TFs MYB, PAX5 and IRF4. Here, we show that hypoSUMOylation leads to enhanced GR occupancy on chromatin and significant changes in gene expression, ultimately sensitizing the B-ALL cells to Dex-induced cell cycle arrest.

## Results

### HypoSUMOylation enhances the ability of glucocorticoid to induce cell cycle arrest of B-ALL cells

**Fig. 1:**
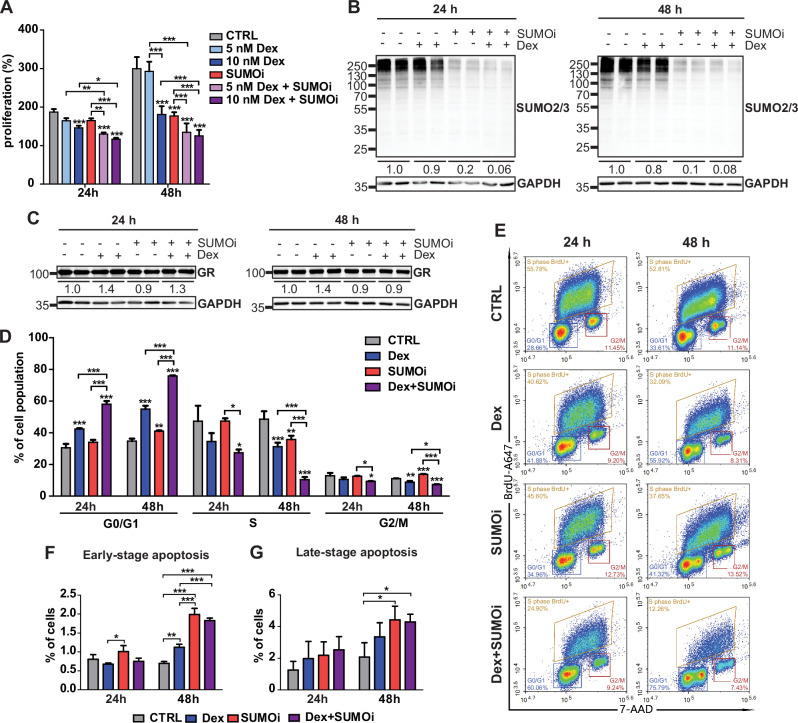
SUMOylation inhibition potentiates Dex-induced cell cycle arrest. **A** Cell proliferation as assessed by MTS assays. Bars represent mean of 5 replicates with SD. Asterisks denote statistical significance calculated with Two-way ANOVA with Bonferroni post hoc test: * = *p* < 0.05, ** = *p* < 0.01, *** = *p* < 0.001. **B** Immunoblotting of SUMO2/3 or (**C**) GR and loading control (GAPDH) in NALM6 cells exposed to vehicle control (DMSO-EtOH), 10 nM Dex, 125 nM SUMOi or combination as depicted by + and – signs. Average SUMO2/3 or GR signal from two replicates normalized to GAPDH and further to time point vehicle control is indicated below the samples. **D** Bar graph of flow cytometric cell cycle analysis. Bars represent mean of 3 replicates with SD. Asterisks denote statistical significance calculated with One-way ANOVA with Bonferroni post hoc test: * = *p* < 0.05, ** = *p* < 0.01, *** = *p* < 0.001. **E** Representative flow cytometry gating strategy for the cell cycle analysis. **F**, **G** Bar graphs of flow cytometric apoptosis analysis for cells in early-stage and late-stage apoptosis stages, respectively. Bars represent mean of 3 replicates with SD. Asterisks denote statistical significance calculated with One-way ANOVA with Bonferroni post hoc test: * = *p* < 0.05, ** = *p* < 0.01, *** = *p* < 0.001.

To assess the sensitivity of NALM6 cells to Dex and SUMOi, we examined their effect on the rate of cell proliferation. Screening with increasing concentrations of Dex (5–100 nM) or SUMOi (125–1000 nM) confirmed their dose-dependent attenuating effect on NALM6 proliferation, with combinations of SUMOi and Dex demonstrating the strongest effects (Supplementary Fig. S1A, B). We then repeated the assay with sub-saturating concentrations of Dex (5 or 10 nM) and SUMOi (125 nM). After 48 h exposure to Dex (10 nM) or SUMOi alone, the rate of NALM6 proliferation nearly halved (181% and 177% vs. 299%, compared to 0 h set as 100%). This reduction in the proliferation rate was already seen at 24 h, although to a lesser extent. Exposure to Dex (10 nM) and SUMOi concomitantly resulted in an even more notable suppression of cell proliferation (125% vs. 299%) (Fig. 1A). Although 5 nM Dex (near the Kd of Dex to GR, [34]) clearly decreased the rate of proliferation when combined with SUMOi, the effect was more evident with 10 nM Dex, which was therefore chosen for further analyses. Evaluation of synergy using HSA reference model implied that Dex and SUMOi act in a synergistic manner (Supplementary Fig. S2A–C). Altogether, the results showed that SUMOi potentiated the cell proliferation-suppressing effect of Dex in NALM6 cells. Moreover, viability assays with another GC-sensitive B-ALL cell line 697, a representative of TCF3-PBX1 fusion, indicated that the potentiating effect of SUMOi on Dex is not restricted to NALM6 cells representing the DUX4-IGH genetic subtype. However, hypoSUMOylation did not render GC-resistant REH cells sensitive to GCs, albeit interestingly, they were more susceptible to SUMOi alone than 697 cells (Supplementary Fig. S3A–C).

To confirm that the SUMOi concentrations were appropriate for achieving hypoSUMOylation, i.e. a suppressed but not completely eradicated level of SUMOylation in the cells, we immunoblotted NALM6 cell extracts with SUMO2/3 antibody after exposure to increasing concentrations of SUMOi. As expected, SUMOi decreased the level of SUMOylation in the cells in a dose-dependent manner (Supplementary Fig. S1C), with 125 nM SUMOi leading to an evidently diminished but not completely erased level of SUMO2/3 conjugation and increased level of free SUMO2/3 (64% decrease and 390% increase, respectively, compared to vehicle control after 24 h exposure) (Supplementary Fig. S1D). Considerably lowered SUMOylation levels in cell extracts were also confirmed after exposure to the combination of 125 nM SUMOi and 10 nM Dex (Fig. 1B). SUMOi did not markedly influence the level of GR in NALM6 cells, causing only a modest (10%) decrease in protein levels compared to vehicle control. Interestingly, after 24 h exposure, treatment with Dex and SUMOi together led to slightly increased (30%) GR protein level (Fig. 1C).

We next investigated the effect of Dex and SUMOi on cell cycle with BrdU labeling of NALM6 cells and flow cytometry. As shown in Fig. 1D, E, the fraction of cells in the S phase decreased with Dex at 48 h compared to the vehicle control, and conversely, the GR agonist increased the number of cells in G0/G1 at 24 h and 48 h. SUMOi also decreased the number of cells in S phase at 48 h and increased the fraction of cells in G0/G1, albeit less efficiently than Dex. Interestingly, SUMOi and Dex together drastically affected the cell cycle phase distribution. Together, they notably increased the fraction of cells in G0/G1 phase (31% → 58% at 24 h and 35% → 76% at 48 h) and reduced the fraction of cells in S phase (47% → 27% at 24 h and 49% → 10% at 48 h) (Fig. 1D, E). The cell cycle thus clearly halted at G0/G1 after 48 h concomitant exposure to SUMOi and Dex.

The actions of GCs in leukemic cells are often attributed to their cytotoxic and apoptosis-inducing effects. To address this, we examined possible changes in the rate of apoptosis in the same experimental conditions with Annexin V assay and flow cytometry. At 24 h, SUMOi marginally increased the number of early-stage apoptotic NALM6 cells, and at 48 h, also Dex increased their amount (Fig. 1F, Supplementary Fig. S1E). However, SUMOi and Dex together showed no additive or synergistic effect on the fraction of early- or late-stage apoptotic NALM6 cells at either time point (Fig. 1F, G, Supplementary Fig. S1E). Moreover, SUMOi did not show clear signs of cytotoxicity, nor did it enhance the modest cytotoxic effect of Dex on NALM6 cells (Supplementary Fig. S1F). Our data thus imply that hypoSUMOylation potentiates proliferation-suppressive effects of Dex in NALM6 cells through the induction of cell growth arrest rather than by increasing the rate of apoptosis.

### Effect of hypoSUMOylation on patient B-ALL cell viability ex vivo

A pharmacologically optimized version of ML-792, TAK-981 has recently demonstrated to enhance Dex sensitivity in MM as well as enhance 5-Aza-2’-deoxycytidine efficacy in B cell lymphoma lines [35–37]. Moreover, as our recent work showed that TAK-981 and ML-792 attenuate prostate cancer cell proliferation to a near-identical degree [38], we decided to assess the sensitivity of patient-derived B-ALL cells to Dex+TAK-981 combination ex vivo. Congruent with our above in vitro results, the combination reduced ex vivo cell viability of several patient samples more effectively than SUMOi (TAK-981) or Dex alone. However, individual responses to Dex, TAK-981 or their combination varied widely between the patients with no simple association with their genetic B-ALL subtype (Supplementary Fig. S4A, B).

### Effect of hypoSUMOylation on GR-regulated genes

**Fig. 2:**
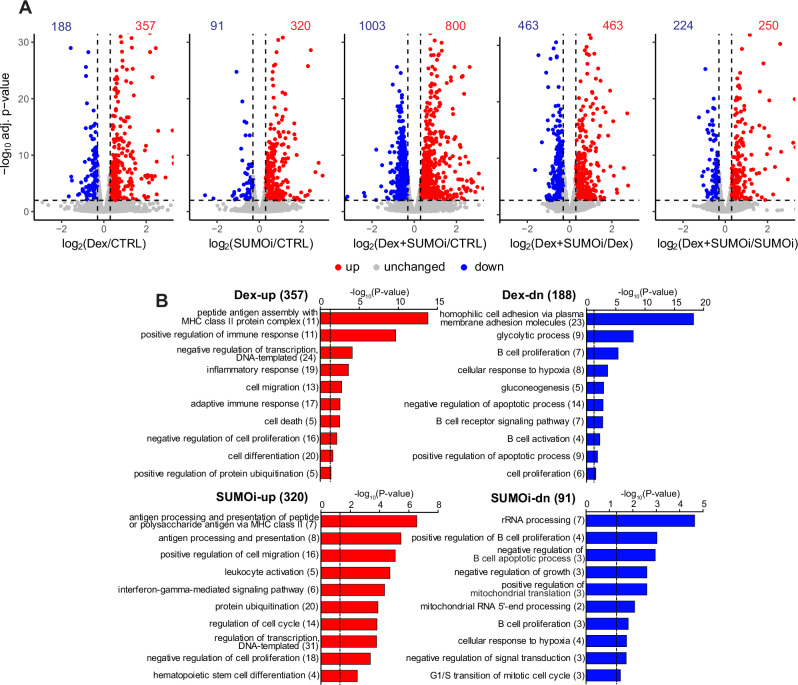
SUMOylation regulates gene expression in NALM6 B-ALL cells. **A** Volcano plots of NALM6 RNA-seq data depicting log_2_[FC] and −log_10_ adjusted *p*-values for pairwise comparisons between treatments. Numbers indicate the number of genes that were upregulated (red, log_2_[FC] > 0.3, −log_10_ adj. *p*-value > 2) and downregulated (blue, log_2_[FC] < 0.3, −log_10_ adj. *p*-value > 2) in response to the treatment. **B** DAVID pathway analyses of Dex-up- or downregulated and SUMOi-up- or downregulated genes. Numbers in parentheses indicate number of genes in pathway. Dashed line indicates −log_10_ transformation of significance threshold (*p*-value < 0.05).

Since the in vitro results indicated that Dex and hypoSUMOylation target the cell cycle, we next analyzed their effect on gene expression in a genome-wide fashion to gain more insight into how the combination of Dex (10 nM) and SUMOi (ML-792, 125 nM) affects cellular processes. Gene expression profiling with RNA-seq revealed that Dex and SUMOi alone regulated the expression of 545 and 411 genes, respectively, and their combination affected the expression of 1803 genes when compared to vehicle control (Fig. 2A). A table of all pairwise comparisons between the treatments is presented in Supplementary Table ST1.

Compared to vehicle control, SUMOi alone upregulated the expression of 320 genes (SUMOi-up) (Fig. 2A, Supplementary Table ST2). The enriched pathways included processes related to antigen processing and presentation, leukocyte activation, and regulation of cell migration, cell cycle and transcription. Among the 91 genes that were downregulated in response to SUMOi (SUMOi-dn) were pathways linked to rRNA processing, mitochondrial translation, and regulation of B cell proliferation and apoptosis. Genes upregulated by Dex alone, compared to vehicle control, (Dex-up; 357 genes) included pathways associated with immune and inflammatory response, as well as transcriptional regulation, cell death and proliferation. Genes downregulated by Dex alone (Dex-dn; 188 genes) were associated with pathways related to glucose metabolism and B cell proliferation, signaling and activation (Fig. 2B) (Supplementary Tables ST2 and ST3).

**Fig. 3:**
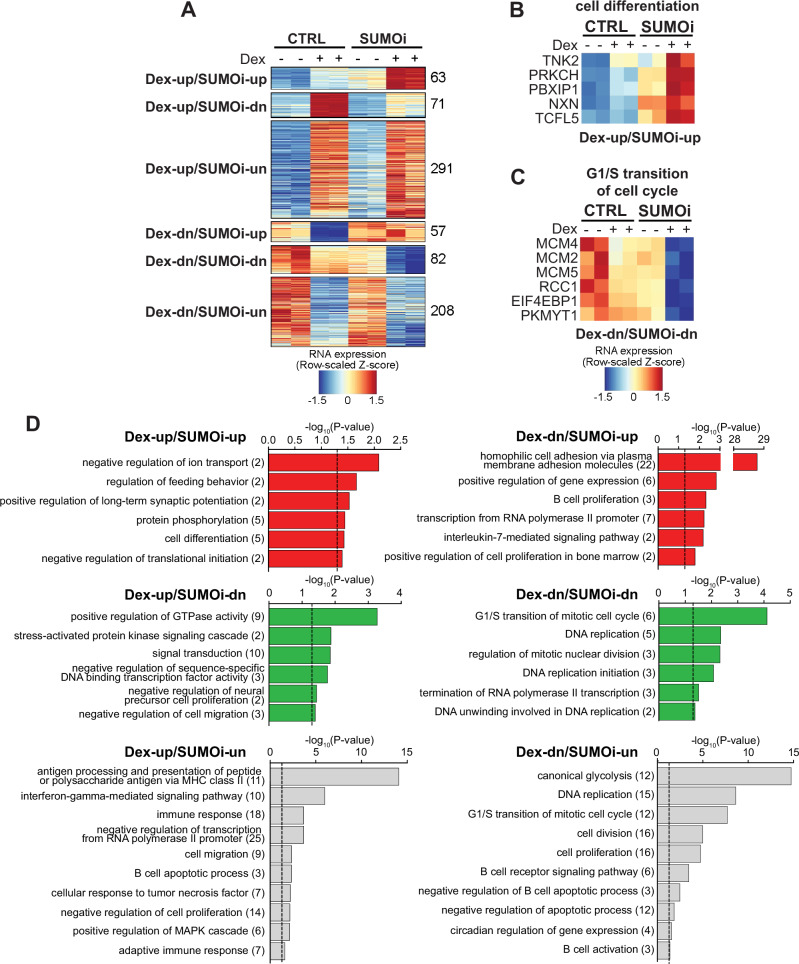
Dex and SUMOi regulate expression of gene subsets in a potentiating manner. **A** Clustering of differentially expressed genes (DEGs) according to Dex vs CTRL and Dex+SUMOi vs Dex effects. Heatmaps of genes in indicated example pathways from DEG clusters Dex-up/SUMOi-up (**B**) and Dex-dn/SUMOi-dn (**C**). **D** DAVID pathway analyses of DEG clusters presented in **A**; numbers in parentheses indicate number of genes in pathway. Dashed line indicates −log_10_ transformation of significance threshold (*p*-value < 0.05).

We next focused on the Dex-regulated genes whose expression was affected in the same direction by both Dex and Dex+SUMOi combination (heatmaps containing all genes behaving in this fashion are presented in Supplementary Fig. S5 and gene lists in Supplementary Table ST3). Clustering of these genes revealed two groups of genes (Dex-up/SUMOi-up and Dex-dn/SUMOi-dn) whose response to Dex+SUMOi combination was interestingly stronger than to either compound alone (Fig. 3A). The group clearly upregulated by Dex+SUMOi (63 genes) included genes, such as TNK2, PRKCH and PBXIP1, involved in cell differentiation and protein phosphorylation (Fig. 3B, Supplementary Table ST3). Notably, the Dex+SUMOi-downregulated group (82 genes) contained genes controlling the G1/S transition of mitotic cell cycle, DNA replication and regulation of mitotic nuclear division (Fig. 3C, Supplementary Tables ST2 and ST3). Although the G1/S transition pathway was also enriched among Dex-downregulated genes unaffected by SUMOi (Dex-dn/SUMOi-un), the genes in the latter group differed from those in the Dex-dn/SUMOi-dn group, with e.g. members of minichromosome maintenance protein complex MCM2, MCM4 and MCM5 belonging solely to the Dex-dn/SUMOi-dn group (Fig. 3C, D, Supplementary Table ST3). This implies that a particular subgroup of G1/S transition associated genes are more sensitive to the combination of Dex and SUMOi. Finding of pathways related to apoptosis in the groups of genes that showed no change with Dex+SUMOi compared to Dex alone (Dex-up/SUMOi-un and Dex-dn/SUMOi-un) is in line with our Annexin V apoptosis assays, where Dex-SUMOi combination did not substantially affect the rate of apoptosis (Fig. 1F, G). In sum, these transcriptomics data indicating gene- and pathway-selective potentiating effect by Dex+SUMOi in cellular processes associated with proliferation are in accordance with our flow cytometry and cell proliferation data.

### HypoSUMOylation augments GR cistrome and modifies the effect of Dex on chromatin accessibility

**Fig. 4:**
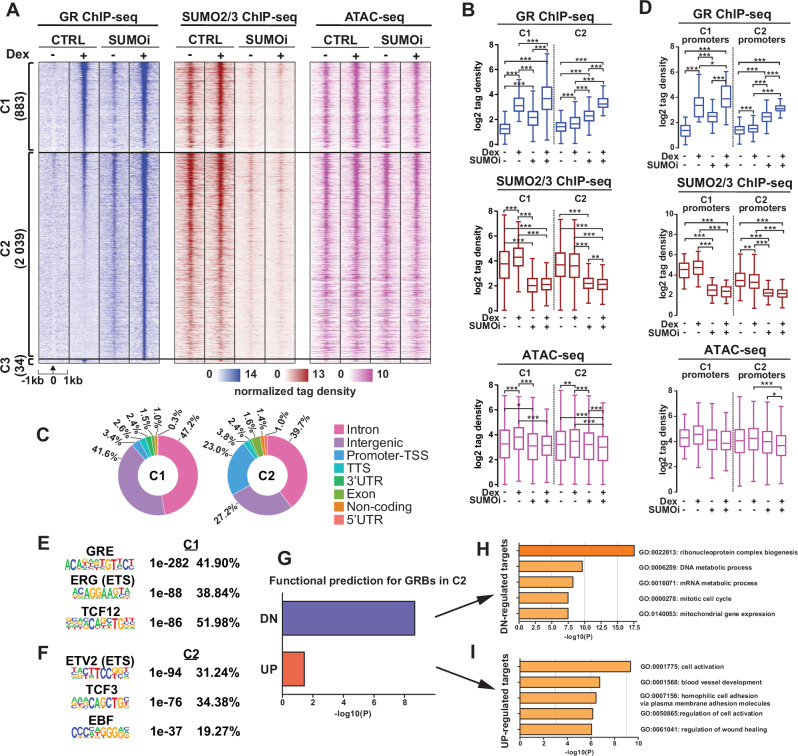
HypoSUMOylation enhances GR binding to chromatin. **A** Heatmaps of GR and SUMO2/3 occupancy on chromatin and chromatin accessibility in moderately SUMOi-affected GRBs (C1, difference between Dex alone and Dex+SUMOi <2-fold) and clearly SUMOi-augmented GRBs (C2, difference between Dex alone and Dex+SUMOi >2-fold). **B** Boxplots of GR and SUMO2/3 ChIP-seq and ATAC-seq signal (tag density) in C1 and C2. **C** Genomic location distribution of GRBs in C1 and C2 clusters. **D** Boxplots of GR and SUMO2/3 ChIP-seq and ATAC-seq signal (tag density) in C1 and C2 promoter regions. **E**, **F** Top three de novo motifs for C1 and C2, respectively, showing *p*-values and percentage of target sites with motif. **G** BETA results for association of C2 GRBs and DEGs (Dex+SUMOi vs Dex). **H** Top 5 Metascape pathways for BETA-predicted direct GR-downregulated target genes. **I** Top 5 Metascape pathways for BETA-predicted direct GR-upregulated target genes. Statistical significance for boxplots was calculated with One-way ANOVA with Bonferroni post hoc test, with asterisks denoting statistical significance: * = *p* < 0.05, ** = *p* < 0.01, *** = *p* < 0.001.

To further examine the crosstalk of GR and SUMOylation, we used ChIP-seq to study the genome-wide binding patterns of GR and SUMO2/3 on chromatin as well as ATAC-seq to assess how Dex and hypoSUMOylation influence chromatin accessibility. Based on SUMO2/3 ChIP-seq, SUMOi indeed overwhelmingly reduced the amount of SUMO2/3 on chromatin, albeit some 2565 chromatin sites (C9) retained a substantial level SUMOylation (Supplementary Fig. S6A, B). As expected, Dex increased the chromatin occupancy of GR compared to vehicle-treated cells. Clustering of Dex-induced GR binding sites (GRBs) according to the effect of SUMOi on them revealed that SUMOi had only a moderate effect (<2-fold difference between Dex and Dex+SUMOi) on the GR occupancy of 883 GRBs (C1, Fig. 4A, B), whereas the occupancy was substantially enhanced (>2-fold) on 2039 sites (C2, Fig. 4A, B). Only 34 sites displayed decreased GR occupancy (log_2_[FC] < −1) in response to SUMOi (C3, Fig. 4A). Genomic coordinates of the GRBs in each cluster are listed in Supplemental Table ST4. SUMOi led to evident hypoSUMOylation in C1 and C2 similarly, without totally erasing SUMO2/3. Dex elevated the level of SUMO2/3 only in C1, albeit the overall SUMO2/3 chromatin occupancy did not markedly differ between C1 and C2 (Fig. 4A, B). In C2, the chromatin occupancy-triggering effect of Dex alone was very modest in comparison to C1, and thus, the C2 sites can be largely considered novel GRBs.

The novel GRBs included for example those localized in or near CBFA2T3 locus whose expression was upregulated in a potentiating manner by the combination of Dex and SUMOi, whereas C1 GRBs included e.g. those in or near FKBP5 locus (Supplementary Fig. S7A–D). Interestingly, SUMOi increased to some extent GR chromatin occupancy both in C1 and C2 even without added Dex (Fig. 4A, B). This may be at least in part be due the presence of residual amount of cortisol (0.51 nM) in the cell culture medium (from the ‘untreated’ FBS). All in all, these data indicate that hypoSUMOylation induces GR chromatin occupancy at sub-saturating GC concentrations, augmenting GR cistrome and creating novel GRBs.

Although chromatin accessibility, i.e. ‘openness’ of most chromatin regions (45743) remained unaffected by Dex and/or SUMOi (C4), Dex exposure influenced the accessibility at 10106 sites, increasing it at 3817 regions (C5 and C6) and decreasing it at 6289 regions (C7 and C8) (Supplementary Fig. S8A, B). De novo motif analysis indicated glucocorticoid response element (GRE) within the top three motifs in C6 but not in C8 (Supplementary Fig. S8C, D). SUMOi blunted the ‘opening’ effect of Dex on chromatin in C6, but not in C5, and enhanced the ‘closing’ effect of Dex in C7 and C8. SUMOi alone increased the chromatin openness in C5 and modestly in C6, whereas it decreased the openness in C7 and C8 (Supplementary Fig. S8A, B). In GR-binding clusters C1 and C2, that showed similar chromatin accessibility, Dex slightly elevated the accessibility and SUMOi similarly blunted the effect of Dex in both clusters (Fig. 4B).

Genomic annotation of GRBs revealed that SUMOi-induced (C2) GRBs were interestingly localized more often on promoter-TSS regions than unaffected (C1) GRBs (23.0% vs. 3.4%) (Fig. 4C). GR and SUMO2/3 occupancy on these promoter sites largely resembled their overall signal in all loci (cf. Fig. 4B, D). Interestingly, de novo motif analysis revealed the GRE as the top motif only within C1 GRBs (Fig. 4E). Instead, the most enriched motif in the new C2 GRBs was that of ETV2, an ETS family member (Fig. 4F). Other enriched motifs in the top three included those of TCF/LEF family members TCF12 in C1 and TCF3 in C2 and that of ERG, an ETS family member in C1, and the binding motif for EBF family of B-cell TFs, in C2 (Fig. 4E, F). Known motif enrichment for GRE also remarkably differed between the two clusters: in C1 GRBs, it was expectedly the top motif (39.30% of sites, *p*-value 1e-334), whereas in C2, it was found only in 4.76% of sites with a much higher *p*-value of 1e-20 (Supplementary Fig. S9A).

Comparison to publicly available H3K4me1, H3K27ac and H3K27me3 ChIP-seq data from NALM6 cells revealed a lower level of H3K4me1 on C2 GRBs than on C1 GRBs (Supplementary Fig. S9B, top panel). The enrichment of H3K27ac, a mark of active promoters and enhancers, and H3K27me3, a repressive mark of bivalent domains, did not differ between the clusters (Supplementary Fig. S9B). The lower enrichment of H3K4me1, a mark of active enhancers, supports the notion of new hypoSUMOylation-induced GRBs being largely localized within promoter regions instead of enhancers.

Binding of B-cell TFs EBF1, IKZF1, PAX5 and RUNX1 were also assessed from published ChIP-seq data. As shown in Supplementary Fig. S9C, C2 GRBs showed clearly lower enrichment of these TFs than C1, with comparison of binding within promoter GRBs showing even drastically lower enrichment in C2 GRBs (Supplementary Fig. S9C). Collectively, these data indicate that hypoSUMOylation augments GR cistrome. In particular, it induces GR chromatin binding to novel sites that are enriched in promoter regions, sparse in GREs, and yet rich in ETS motifs. To a limited extend and in a locus-selective fashion, hypoSUMOylation also modifies the Dex-induced alterations on chromatin accessibility. However, the latter alterations alone cannot simply explain the augmenting effect of hypoSUMOylation on GR chromatin occupancy and reprogramming of the receptor cistrome.

### GR represses gene expression from its novel hypoSUMOylation-induced binding sites

We then applied Binding and Expression Target Analysis (BETA) to associate the new GRBs with genes that had been regulated by the Dex+SUMOi in a potentiating manner (Dex-up/SUMOi-up and Dex-dn/SUMOi-dn, Fig. 3A) in the transcriptome data. BETA considers both the regulatory potential of TF binding, in this case GR, using a distance-weighted function to gauge the contribution of individual binding sites within ±100 kb of target gene TSS and the differential expression upon TF binding to predict direct target genes and determine the overall regulatory effect, whether the TF has activating (upregulation) or repressive (downregulation) function [39]. All BETA data from different treatment comparisons from C1 and C2 clusters are presented in Supplementary Fig. S10. The association data revealed that on the new hypoSUMOylation-induced GRBs (C2), GR predominantly had a repressive function on gene expression (Dex+SUMOi vs. Dex; *p* < 2.14e-09, Fig. 4G). Contrastingly, in other comparisons (e.g. with Dex alone in C1 GRBs), GR chiefly had an activating function on gene expression (Supplementary Fig. S10A–C). Enrichment analysis with Metascape [40] revealed genes associated with mitotic cell cycle among the predicted GR-down-regulated target genes in C2, while upregulated target genes in the cluster included those associated with cell activation (Fig. 4H–I). GR-regulated suppression of genes linked to cell cycle is consistent with our transcriptome and cell cycle data.

### Chromatin-protein network of GR is interlinked with SUMO2/3 in NALM6 cells

**Fig. 5:**
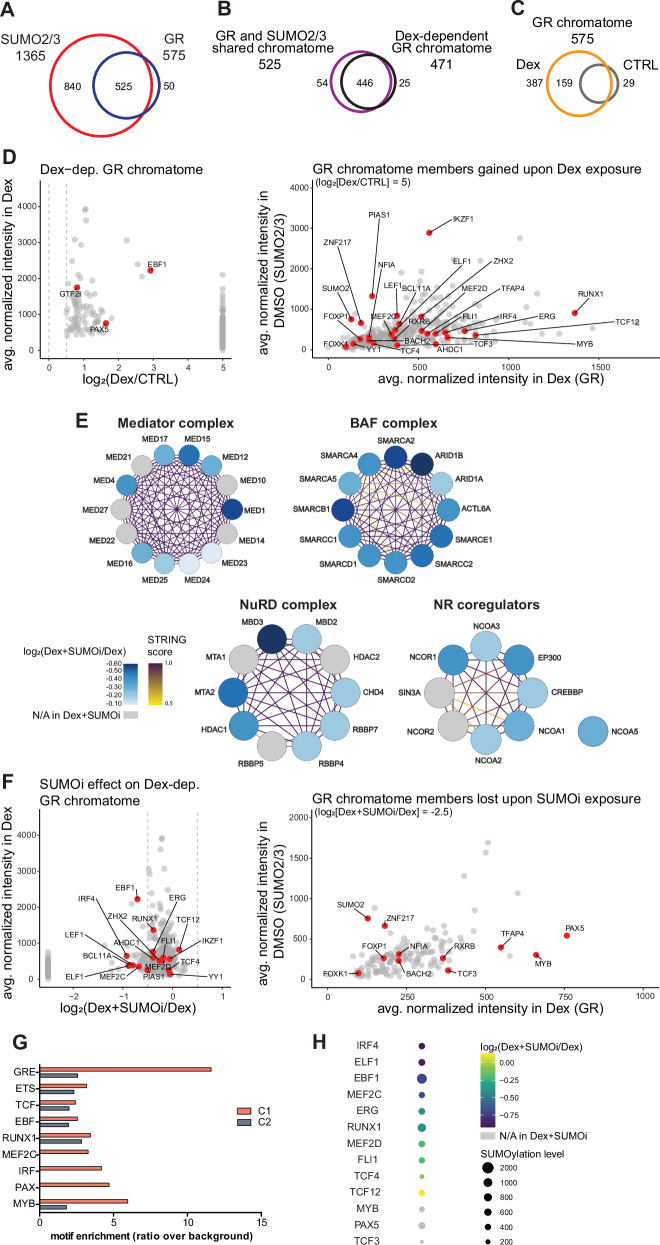
SUMOylation modulates the composition of GR chromatome in NALM6 cells. **A** Venn diagram of the overlap of RIME-identified SUMO2/3 chromatome and GR chromatome. **B** Venn diagram of the overlap of RIME-identified GR and SUMO2/3 shared chromatome and Dex-dependent GR chromatome. **C** Venn diagram of RIME-identified GR chromatome in the presence and absence of Dex. **D** Scatterplot on the left depicts the Dex-dependent (log_2_[Dex/CTRL] ≥ 0.5) proteins associated with GR. Proteins found associated with GR only in Dex (gained upon Dex) are set to have a log_2_(Dex/CTRL) = 5 and are presented in the right panel as normalized intensities in Dex (GR) plotted against normalized intensities in DMSO (SUMO2/3). TFs are highlighted in red. **E** Coregulator complexes identified in GR chromatome. Blue color scale shows log_2_(Dex+SUMOi/Dex) and purple-yellow color scale indicates the confidence of interaction according to the STRING database. NR, nuclear receptor. **F** Scatterplot of the effect of SUMOi on the Dex-dependent chromatome of GR. Left panel depicts the Dex-dependent (log_2_[Dex/CTRL] ≥ 0.5) proteins associated with GR. Proteins found associated with GR only in Dex are set to have a log_2_(Dex+SUMOi/Dex) = −2.5 and are presented in the right panel as normalized intensities in Dex (GR) plotted against normalized intensities in DMSO (SUMO2/3). TFs are highlighted in red. **G** Fold enrichment bar graph for de novo TF binding motifs in GRB clusters C1 and C2 with cognate proteins found in GR chromatome. **H** Dot plot showing SUMOi-effect (log_2_[Dex+SUMOi/Dex]) on TFs with cognate binding motifs presented in **G**. Size of dot corresponds to SUMOylation level of the protein by showing average normalized intensity in DMSO (SUMO2/3).

To investigate the composition of the chromatin-protein networks, chromatomes, of GR and SUMO2/3 in NALM6 cells, we used a RIME-based approach (see Materials and Methods), which employs affinity purification of endogenous chromatin-protein complexes and subsequent mass spectrometry analysis for identifying proteins surrounding the chromatin-bound factor. This led to identification of 575 chromatin-bound GR-associated proteins and 1365 SUMO2/3-associated proteins (Fig. 5A, Supplementary Table ST5). The vast majority of GR-associated proteins (525 out of 575) were also found in the chromatome of SUMO2/3, implying that the chromatin partners of GR are principally SUMOylated (Fig. 5A, Supplementary Table ST5).

The GR chromatome was Dex-dependent to a great degree, as 471 proteins showed increased association (log_2_[FC] ≥ 0.5) with GR following Dex treatment (Fig. 5B, D, left panel). A large portion of the Dex-dependent chromatome (387 proteins), including SUMO2 and SUMO E3 ligase PIAS1, were associated with GR solely after Dex induction (Fig. 5C, D, right panel). Transcriptional coregulators represented half of the proteins (49.7%) within the Dex-dependent chromatome. These coregulators included 14 members of the mediator complex (MED1, -4, -10, -12, -14, -15, -16, -17, -21, -22, -23, -24, -25 and -27), 12 components of BAF complex (ACTL6A, ARID1A, ARID1B, SMARCA2, SMARCA4, SMARCA5, SMARCC1, SMARCC2, SMARCB1, SMARCD1, SMARCD2 and SMARCE1), members of NuRD complex (CHD4, HDAC1, HDAC2, MTA1, MTA2, MBD2, MBD3, RBBP4, RBBP5 and RBPP7), nuclear receptor coactivators, NCOA1, NCOA2, NCOA3, and NCOA5, coactivators-cointegrators, EP300 and CREBBP, and corepressors, NCOR1, NCOR2 and SIN3A. Interestingly, 5 members of the MCM complex (MCM3, -4, -5, -6 and -7) were also among the Dex-dependent chromatome (Fig. 5E and Supplementary Fig. S11A–E, Supplementary Table ST5).

### B-cell transcription factors co-occupy chromatin with GR

The Dex-dependent GR chromatome also included a considerable number of TFs. These 27 TFs included EBF1, ELF1, ERG, FLI1, FOXK1, FOXP1, GTF2I, IKZF1, IRF4, LEF1, MEF2C, MEF2D, MYB, PAX5, RUNX1, TCF3, TCF4 and TCF12 (Fig. 5D). Practically all these TFs and above mentioned coregulators were also found in SUMO2/3 chromatome (96.3% and 91.5%, respectively) (Supplementary Table ST5). We next compared the identified TF composition of Dex-induced GR chromatome with their de novo binding motif information from GRBs identified in the ChIP-seq data. For several TF binding motifs, the cognate TFs, EBF1, ELF1, ERG, FLI1, IKZF1, IRF4, MEF2C, MEF2D, MYB, PAX5, RUNX1, TCF3, TCF4, and TCF12, were found in GR’s chromatome (Fig. 5D, G, H). Our data thus indicate that GR and these TFs, many of which have essential functions in B-cells, reside on chromatin closely in parallel (within 200 bp).

### HypoSUMOylation attenuates the association of GR with its chromatin partners

Given the major overlap between the chromatomes of GR and SUMO2/3, we next assessed the impact of hypoSUMOylation on the Dex-dependent chromatome. Inhibition of SUMOylation had a generally decreasing effect on the association strength of GR chromatome members. Interestingly, 144 proteins lost their association with GR completely upon hypoSUMOylation. This group included TFs, such as TCF3, PAX5, FOXP1, FOXK1 and MYB, and coregulators, such as HDAC2, MTA1, RBBP5, NCOR2 and SIN3A, and members of the mediator complex (MED10, -14, -21, -22, -27) (Fig. 5E, F). As expected, association of SUMO2 with GR was also lost with SUMOi treatment (Fig. 5F, right panel). Moreover, for example, TFs EBF1, IRF4, ELF1, MEF2C and LEF1 showed a decreased association with GR after treatment with Dex+SUMOi combination compared to Dex alone (log_2_[FC] ≤ −0.5) (Fig. 5F, H). Interestingly, motifs for PAX(5), MEF2C and IRF(4) were found only in C1, indicating their absence near or at the novel SUMOi-induced GRBs (Fig. 5G). The motif for MYB was also considerably less enriched in C2. Thus, based on our data, SUMOylation may directly or indirectly regulate the interactions of several coregulators as well several TFs, including IRF4, PAX5, TCF3, MYB, EBF1, ELF1 and LEF1 with GR and/or chromatin.

## Discussion

Synthetic GCs, especially Dex and prednisolone, play a crucial role in the treatment of ALL, a prevalent and aggressive pediatric cancer. While GCs have been foundational in the treatment regimen, their efficacy is hampered by resistance and adverse side effects [4]. GCs signal is mediated through GR whose regulatory function in cell growth is modulated by SUMOylation [17, 18]. Recent studies have identified SUMOylation as a potential therapeutic target in hematological malignancies such as AML and MM [11–14, 24, 27, 35, 37].

Here, we have elucidated the relationship between SUMOylation and GR action in the context of B-ALL where the role of SUMOylation has remained elusive. To that end, we employed unbiased genome-wide approaches and chromatin-directed proteomics with a B-ALL cell model in conjunction with cell growth and cell cycle assays. Our results show that the combination of hypoSUMOylation and sub-saturating GC treatment more effectively suppressed cell proliferation in NALM6 B-ALL cells than either treatment alone, primarily through induction of cell cycle arrest rather than enhancing apoptotic pathways. This suggests a synergistic effect of hypoSUMOylation and GC treatment on growth suppression, shifting the therapeutic effect away from cytotoxicity towards a potentially less deleterious mechanism of action. The therapeutical effects of supraphysiological levels of GCs are thought to be primarily attributed to their ability to induce leukemic cell death, notably by repressing antiapoptotic BCL2 and simultaneously activating proapoptotic BIM [41]. Recent studies with NALM6 cells have proposed that multiple factors contribute to the cytotoxic effects of GCs, with also GC-increased glycolysis-caused depletion of energy stores and GC-dependent suppression of B-cell development genes being linked to therapeutic response [28, 31, 42].

Our findings indicate that hypoSUMOylation significantly enhances GR occupancy on chromatin, leading to an altered gene expression profile that particularly affects cell cycle regulation. These results are in line with our data from a heterologous HEK293 cell model showing that both mutation of GR SUMOylation sites and SUMOi enhance GR chromatin occupancy and affect expression of genes associated with cell growth and proliferation processes [17, 18]. The augmented GR chromatin occupancy in hypoSUMOylated B-ALL cells revealed an expanded cistrome, with novel GRBs that are enriched in promoter regions and characterized by a distinct TF motif landscape. Although hypoSUMOylation to certain extent also altered chromatin accessibility, these alterations alone cannot explain the augmenting effect of hypoSUMOylation on GR chromatin occupancy and reprogramming of the receptor cistrome. Intriguingly, the novel hypoSUMOylation-induced GRBs were practically devoid of GRE motifs, whereas they were clearly enriched in other unaffected GRBs, being the top motif among them. Instead, the top motif among the hypoSUMOylation-induced GRBs was that of an ETS family TF, ETV2. However, the *p*-value of ETV2 was relatively moderate and its frequency of occurrence was even lower than that of another ETS family member (ERG) in the GRBs unaffected by hypoSUMOylation. Nonetheless, members of ETS family, including ERG and FLI1, have been found to interact with GR in a heterologous system, with the latter one augmenting the receptor’s transcriptional activity [43]. Interestingly, ETS factors have been shown to markedly enhance the chromatin occupancy of androgen receptor and reprogram its cistrome in prostate cancer cells [44], resembling the effect of hypoSUMOylation on GR cistrome in B-ALL cells. Notably, the emergence of these new hypoSUMOylation-induced GRBs was associated with the downregulation of genes involved in the G1/S transition of the cell cycle and DNA replication, which likely contributes to the observed hypoSUMOylation-induced enhancement of GC-induced growth arrest.

Chromatin-directed proteomics enabled us to identify the chromatomes of GR in B-ALL cells, which for the first time provides a comprehensive and unbiased insight into the transcriptional coregulators and complexes as well as TFs intimately collaborating with GR in leukemia cells. The network that is highly Dex-dependent includes several coregulators, e.g. CREBBP, NCOR1, TBL1XR1 and ARID1A, and B cell lineage TFs, e.g. RUNX1, EBP1, ERG, IKZF1, LEF1, PAX5, TCF3, TCF4 and TCF12, whose genes are frequently disorganized in B-ALL [45]. The co-occupancy of B-cell TFs with GR on chromatin underscores a concerted action of these TFs in regulating gene expression that likely defines the specificity and efficacy of GR-mediated transcriptional regulation in B-ALL cells. Comparison of these NALM6 cell proteomics data with our and others recent GR proteomics data from human epithelial lung carcinoma (A549) and embryonic kidney (HEK293) cells reveals that the coregulator interaction profiles of Dex-activated GR are similar (including all e.g. mediator complex members, nuclear receptor coregulators, nuclear receptor corepressors, and members of BAF complexes) and yet the TF interaction profiles of GR considerably differ between the cell lines [18, 46]. This highlights the cell-type specificity of the TF segment of GR chromatome and the variety of the receptor’s potential crosstalk partners in different GC target tissues, supporting the importance of GR’s crosstalk with TFs in conferring tissue-specificity in GR-regulated gene expression. The considerable (44%, Supplementary Fig. S12) overlap of GR chromatome with recently identified coregulators contributing to GC sensitivity of B-ALL cells [28] also points to the biological relevance of the identified GR chromatome which is anticipated to serve as an important resource for forthcoming research.

Furthermore, our chromatome analysis underscores the extensive overlap between GR-associated and SUMO2/3-associated chromatin proteins, strongly suggesting that the majority of GR chromatome members are subject to SUMOylation. The observed decrease or loss in the association of key B-cell TFs, e.g. EBF1, IRF4, ELF1, LEF1, TCF3, PAX5 and MYB, and coregulators, e.g. HDAC2, NCOR2, GATAD2B, with GR upon hypoSUMOylation implies that SUMOylation is directly or indirectly involved in regulating the genomic targeting of GR and the formation and/or stability of GR-containing transcriptional complexes. Given the critical roles of these TFs play in B-cell development and function, their altered interaction with GR under hypoSUMOylated conditions could be pivotal in redirecting leukemic cell fate towards growth arrest.

In conclusion, our study provides compelling evidence that targeting SUMOylation can potentially enhance therapeutic action of glucocorticoids in ALL by modifying GR chromatin occupancy and its interaction with transcriptional networks. This approach may not only offer a potential strategy to alleviate GC resistance by lowering the therapeutic GC dose but also open avenues for more selective and less toxic therapeutic interventions, possibly also for other cancers where GCs are a component of the therapeutic regimen. Our ex vivo results also imply that targeting SUMOylation may have potential in enhancing current B-ALL therapies. However, further in vivo studies are needed to validate these notions.

## Methods

### Antibodies

Anti-GR (sc-1003, used for ChIP-seq), normal mouse IgG (sc-2025), and anti-GAPDH (sc-25778) were from Santa Cruz Biotechnology (Santa Cruz, CA, USA); anti-SUMO2/3 antibodies were from MBL International Corporation (M114-3; Woburn, MA, USA) and Zymed (51-9100, San Francisco, CA, USA); anti-GR (D6H2L #12041, used for RIME) was from Cell Signaling Technology (Danvers, MA, USA); normal rabbit IgG (12-370) was from Merck Millipore (Burlington, MA, USA); anti-BrdU A647 was from Biolegend (#370704, San Diego, CA, USA).

### Cell culture and compounds

Human pre-B cell line NALM6 (ACC-128, DSMZ, Germany) was maintained in Roswell Park Memorial Institute (RPMI) 1640 medium supplemented with 2 mM L-glutamine (Gibco, Waltham, MA, USA), 1 U/µl penicillin, 1 µg/ml streptomycin (Gibco #15140-122) and 10% (v/v) fetal bovine serum (FBS) (Gibco Performance #26140-079). FBS contained 5.1 nM cortisol as analyzed with SCIEX Triple Quad 7500 LC-MS/MS system (The Turku Metabolomics Center/Turku Bioscience Centre). REH and 697 cells were cultured in RPMI media (Gibco) supplemented with 2 mM L-glutamine (Gibco), 1% antibiotics (0.5 U/ml penicillin and 0.5 μg/ml streptomycin; Lonza, Basel, Switzerland), and 10% (v/v) FBS (Gibco). All cells were routinely tested for mycoplasma contamination. For experiments, cells were treated with indicated concentrations of dexamethasone (Dex, Sigma-Aldrich, St. Louis, MO, USA) and ML-792 (SUMOi, MedKoo Biosciences #407886, Morrisville, NC, USA).

### Proliferation and cytotoxicity assays

Cell proliferation was determined using CellTiter 96 AQueous One Solution Cell Proliferation Assay (Promega #G3580, Madison, WI, USA) according to the manufacturer’s protocol. Cells were seeded into 96-well plates at a density of 3 × 10^4^ cells/well, in 5 replicates, and treated with vehicle (DMSO-EtOH), Dex, SUMOi or combination as indicated, in a total volume of 200 µl/well. 40 µl of CellTiter reagent was added to each well and incubated for 3 h prior to measuring absorbance at 492 nm. Statistical significance was determined with Two-way ANOVA with Bonferroni post hoc test. Synergy between Dex and SUMOi was calculated based on Highest Single Agent (HSA) reference model [47] using SynergyFinder Plus R package [48] with R-studio version 2023.06.1 and R v4.3.1. Cytotoxicity was measured using CytoTox 96 Non-Radioactive Cytotoxicity Assay kit (Promega) following the manufacturer’s instructions. Cells were cultured in RPMI-1640 medium with 5% FBS, 1% (v/v) L-glutamine, and 1 U/µl penicillin and 1 µg/ml streptomycin. Cytotoxicity was calculated as a percentage of maximum LDH release control, according to manufacturer’s protocol.

### Cell cycle and apoptosis assays

Phase Flow Alexa Fluor 647 BrdU Kit (Biolegend #370704) was used for cell cycle analysis. NALM6 cells were treated with vehicle (DMSO-EtOH), 10 nM Dex, 125 nM SUMOi or combination in triplicate for 24 h and 48 h, and then pulsed with 5 µg/ml BrdU for 1 h at 37 °C, 5% CO_2_ before harvesting. 1 × 10^6^ cells were stained with anti-BrdU A647 antibody and 7-AAD according to manufacturer’s instructions prior to acquiring on a flow cytometer. For apoptosis assays, 1 × 10^6^ cells were stained with APC Annexin V Apoptosis Detection Kit with 7-AAD (Biolegend #640930) according to manufacturer’s instructions. Samples were analyzed with NovoCyte Quanteon flow cytometer (Agilent, Santa Clara, CA, USA) using NovoExpress software (Agilent). In flow cytometry analysis, NALM6 cells were analyzed according to morphological properties using FSC-A and SSC-A parameters. Next, single cells were gated according to FSC-A and FSC-H parameters. For cell cycle analysis, the cells were next assigned into G0/G1, G2/M and S-phases according to the distribution of cells using BrdU A647 and 7-AAD stainings, and with help of staining controls without BrdU A647 staining. For the apoptosis assay analysis, the cells were analyzed as early-stage apoptotic cells (Annexin V+, 7-AAD−) or as late-stage apoptotic cells (Annexin V+, 7-AAD+) according to the distribution of cells using Annexin V APC and 7-AAD stainings, and with help of staining controls without 7-AAD staining. Statistical significance was determined with One-way ANOVA with Bonferroni post hoc test.

### Ex vivo drug response assays

Mononuclear cells (MNCs) of pediatric B-ALL samples were used. The study was approved by the Regional Ethics Committee in Pirkanmaa, Tampere, Finland (#R13109), conducted according to the guidelines of the Declaration of Helsinki with a written informed consent received by the patient and/or guardians. The MNCs were extracted from fresh bone marrow using the density gradient centrifugation with Ficoll-Paque Plus (GE Healthcare, Helsinki, Finland, #17-1440-02) or LymphoPrep^TM^ (StemCell Technologies, Vancouver, Canada). MNCs were viably frozen in 15% DMSO/40% FBS in RPMI in liquid nitrogen. For drug treatments, MNCs were thawed using an in-house method, and 2 × 10^4^ cells/well were plated on 96-well plates. Cells were treated with TAK-981 (100 nM; #S8829, Selleckchem, Houston, TX, USA) or Dex (10 or 25 nM) alone or with TAK-981+Dex combination at 37 °C in 5% CO_2_, and cell viability was measured after 48 h using CellTiter-Glo assay (Promega). The results were normalized using DMSO-treated cells to obtain relative viability.

### Immunoblotting

NALM6 cells were treated with vehicle (DMSO-EtOH), 10 nM Dex, 125 nM SUMOi or combination for 24 h and 48 h, or with DMSO and increasing concentrations of SUMOi as indicated, and then pelleted by centrifugation and washed with ice-cold TBS supplemented with 20 mM N-ethylmaleimide and protease inhibitor cocktail (PIC, Roche, Basel, Switzerland). Cells were suspended in SDS-PAGE sample buffer with PIC and 10 mM N-ethylmaleimide and heated at 95 °C for 2 min, followed by lysis by sonication (2 × 10 sec). Lysates were supplemented with 2% β-mercaptoethanol and reheated at 95 °C for 2 min before separation on 10% SDS-PAGE gels, or gradient SDS-PAGE gels (4–20% Mini-Protean TGX Precast Protein Gels, Bio-Rad 4561093, Hercules, CA, USA) for blotting free, unconjugated SUMO2/3. Proteins were transferred onto nitrocellulose or PVDF membranes and probed by antibodies against SUMO2/3 (MBL, M114-3 and Zymed, 51-9100) GR, and GAPDH (Santa Cruz Biotechnology, sc-25778). Appropriate horseradish peroxidase-conjugated secondary antibodies (Life Technologies, Carlsbad, CA, USA) and chemiluminescence reagent (Pierce, Waltham, MA, USA) were used for detection.

### RNA-seq

Protocol was modified from ref. [18]. Briefly, NALM6 cells were seeded onto 6-well plates and exposed to vehicle (DMSO-EtOH), Dex (10 nM), SUMOi (125 nM) or combination for 24 h, in biological triplicates. Total RNA was extracted with Monarch Total RNA Miniprep Kit (T2010S, New England Biolabs, Ipswich, MA, USA) and mRNA isolated with NEBNext Poly(A) mRNA Magnetic Isolation Module (E7490, New England Biolabs) according to manufacturer’s recommendations. RNA-seq libraries were generated with NEBNext Ultra II Directional RNA library prep kit (E7765, New England Biolabs) according to manufacturer’s recommendations. The sequencing data were processed using an in-house pipeline. Trimmed raw reads were aligned to human genome assembly GRCh38 (hg38) from Genome Reference Consortium using STAR [49]. Total count per gene was calculated using TPM normalization. Genes with TPM > 0.5 in at least one sample in any condition were considered expressed. Differentially expressed genes were defined as those with adjusted *p*-value of <0.01 and log_2_(fold change [FC]) of <−0.3 or >0.3. Differentially expressed gene sets were analyzed with DESeq2 using HOMER [50]. Downstream RNA-seq analysis was performed as described [51]. Differentially expressed gene sets were subjected to pathway analysis with DAVID functional annotation tool (GOTERM_BP_DIRECT) [52].

### ATAC-seq

NALM6 cells were seeded in 6-well plates 1 × 10^6^ cells/well and treated with vehicle (DMSO-EtOH), Dex (10 nM), SUMOi (125 nM) or combination for 24 h. Nuclei isolation was performed as described in ref. [18] with the following modifications: harvested, pelleted cells were washed with ice-cold PBS buffer supplemented with PIC. The pellet was resuspended in ice-cold Buffer A (15 mM Tris-HCl, pH 8.0, 15 mM NaCl, 60 mM KCl, 1 mM EDTA, pH 8.0, 0.5 mM EGTA, pH 8.0, 0.5 mM spermidine, PIC in sterile H_2_O; modified from ref. [53]) to a final concentration of 6 × 10^6^ cells/ml. To isolate nuclei, an equal volume of Buffer A supplemented with 0.04% (v/v) IGEPAL CA-630 was added, to achieve a final concentration of 3 × 10^6^ cells/ml and 0.02% (v/v) IGEPAL. After incubation on ice for 10 min, the nuclei were pelleted, washed twice with ice-cold Buffer A and twice with ATAC-Resuspension Buffer (10 mM NaCl, 10 mM Tris-HCl, pH 7.4, 3 mM MgCl_2_ in sterile H_2_O). The isolation of nuclei was verified with Trypan blue counting. Transposition reaction and PCR amplification were performed with 100 000 nuclei according to the published protocol [54] with minor modifications. Transposition reaction was incubated at 37 °C, 800 rpm, for 45 min. Transposed DNA was purified immediately using Monarch PCR & DNA Cleanup Kit (T1030, New England Biolabs) according to manufacturer’s instructions. For the first PCR amplification, the following thermal cycle settings were used: 1 cycle of 75 °C for 5 min, 98 °C for 30 sec; 5 cycles of 98 °C for 10 sec, 63 °C for 30 sec, 72 °C for 1 min. The second PCR amplification was performed with a reaction volume of 20 µl, containing 5 µl of previously PCR amplified DNA, 2.5 µl 12.5 µM PCR Primer 1, 2.5 µl 12.5 µM barcoded PCR Primer 2, 10 µl LightCycler 480 SYBR Green Master I reaction mix (Roche). Primer sequences are available in ref. [54]. The number of additional cycles needed for each sample was calculated as in ref. [54] by determining the cycle number that corresponded to 1/3 of the maximum fluorescent intensity. Size selection with SPRIselect beads (Beckman Coulter, Brea, CA, USA) was performed according to the manufacturer’s instructions to remove fragments <150 bp and >800 bp. Quality of the purified libraries was assessed using a Bioanalyzer High Sensitivity DNA Analysis kit (5067-4626, Agilent) according to the kit instructions. Prior to sequencing, the purified DNA was stored in DNA LoBind Tubes (Eppendorf, Hamburg, Germany). Based on the Bioanalyzer results, the best libraries were pooled and sent for sequencing with Illumina NextSeq 500 (40PE). Two biological replicates were sequenced in the EMBL Genomics Core Facility (Heidelberg, Germany).

### ChIP-seq

Chromatin immunoprecipitation (ChIP) was performed as described in ref. [17] with the following modifications. A total of 30–40 x 10^6^ NALM6 cells per condition were treated with vehicle (DMSO-EtOH), Dex (10 nM), SUMOi (125 nM) or combination for 24 h. Cells were crosslinked with 1% formaldehyde for 8 min, crosslinking was quenched with 125 mM glycine for 8 min, and cells were lysed twice in Farnham Lysis Buffer on ice. Chromatin was fragmented to an average size of 150–200 bp by sonication (Bioruptor, UCD-300, Diagenode, Liege, Belgium), with 3–4 × 10^6^ cells per sonication aliquot. Antibodies were coupled to magnetic protein G beads (Dynabeads, Invitrogen, Waltham, MA, USA) for 16 h, and after pooling the sonication aliquots back together the lysates were then incubated with antibody-coupled beads for 16 h. Antibodies used per IP: GR (sc-1003, Santa Cruz), 2 μg; SUMO2/3 (M114-3, MBL), 2 μg. 5 IP samples were pooled for one ChIP-seq sample. ChIP-seq libraries were prepared using NEBNext Ultra II DNA Library Prep Kit (E7103, New England Biolabs) according to manufacturer’s protocol. Two biological replicate samples were sequenced using NextSeq 500 (75SE).

### ChIP-seq and ATAC-seq data analysis

ATAC-seq read filtering was performed as previously described [17, 55]. Subsequently, paired-end samples were aligned to hg38 genome using Bowtie2 [56]. Alignment was performed with end-to-end sensitive mode allowing no mismatches. Downstream data analysis was performed using HOMER [50]. Peaks in each dataset were called using findPeaks with style factor, FDR < 0.01, >25 tags, >6-fold over local background. Subsequently, hg38 ENCODE blacklist peaks were filtered out. getDifferentialPeaks was used to isolate differential binding peaks (Poisson *p*-value < 0.0001, FC > 3) between the different treatment conditions. Aggregate plots and heatmaps were generated with 10 bp or 20 bp bins surrounding ±1 kb area around the centre of the peak. All plots were normalized to 10 million mapped reads and further to tags per site per bp. De novo motif searches were performed using findMotifsGenome.pl with the following parameters: 200 bp peak size window, strings with 2 mismatches, binomial distribution to score motif *p*-values, and 50,000 background regions.

ChIP-seq data analysis was performed as previously described [17, 55] and downstream data analysis was performed using HOMER [50]. Peaks in each dataset were called using findPeaks with style factor, FDR < 0.001, >25 tags, >4-fold over control sample and local background. For GR ChIP-seq, vehicle (DMSO-EtOH) treated cells were used as control; for SUMO2/3 ChIP-seq, ChIP input sample was used as control sample. getDifferentialPeaks was used to isolate differential binding peaks (Poisson *p*-value < 0.0001, FC > 3; for GR peaks, FC > 2) between different treatment conditions. Aggregate plots and heatmaps were generated with 10 bp or 20 bp bins surrounding ±1 kb area around the centre of the peak. All plots were normalized to 10 million mapped reads and further to local tag density, tags per bp per site. Box plots represent log_2_ tag counts. GRBs were categorized into cluster (C)1 and C2 based on FC, with C1 representing GRBs where the difference between Dex alone and Dex+SUMOi combination was <2-fold (−1 < log_2_[FC] < 1), and C2 representing those with >2-fold difference (log_2_[FC] > 1). De novo motif searches were performed using findMotifsGenome.pl as described above. Statistical significance was determined with One-way ANOVA with Bonferroni post hoc test. Integration of ChIP-seq, ATAC-seq and RNA-seq data was performed using BETA software [39]. Pathway analyses for predicted GR target genes were performed using Metascape (GO Biological Processes) [40]. For comparing de novo motifs with GR-associated proteins on chromatin, known motif matches to de novo motifs with a HOMER comparison score of ≥0.6 were considered when associating motifs with cognate proteins.

### Chromatin-directed proteomics (RIME)

Rapid immunoprecipitation mass spectrometry of endogenous proteins (RIME) was performed as described in [57] with the following modifications. NALM6 cells were seeded 22.5 × 10^6^ cells per flask in maintenance medium as three biological replicates and the cells were treated with vehicle, Dex, SUMOi or combination for GR RIME, and with DMSO vehicle or SUMOi for SUMO2/3 RIME, as described above. Briefly, crosslinking and cell lysate preparation were performed as in ChIP-seq experiments described above. Fragmented chromatin was incubated with antibody overnight (16 h) at 4 °C, in a total volume of 1 ml RIME-RIPA buffer (prepared in LC-MS grade H_2_O). Antibodies used per chromatin-antibody coupling: GR (Cell Signaling Technology), 12.5 µl; normal rabbit IgG (Millipore), 2 µg; normal mouse IgG (Santa Cruz), 2 µg; SUMO2/3 (MBL), 2 µg. Magnetic beads were blocked and washed with buffers prepared in LC-MS grade H_2_O as described above. Antibody-coupled chromatin was collected on beads in 15 mL Protein LoBind tubes (Eppendorf) over 16 h at 4 °C: five chromatin aliquots originating from the same sample were pooled during IP. Washes with RIME-RIPA buffer and 100 mM ammonium hydrogen carbonate were performed as described in [57]; samples were transferred into new 1.5 mL Protein LoBind tubes (Eppendorf) after first RIME-RIPA wash. Beads were then flash frozen and analyzed with high resolution mass spectrometry following the original RIME protocol [57]. Enzymatic digestion, peptide de-salting by solid-phase extraction and LC-MS/MS were performed as described in [57]. Briefly, beads were washed with ammonium bicarbonate and sequencing grade modified trypsin was added to the beads for 15 min with frequent vortexing and left for digestion for overnight in 37 °C without further agitation, followed by added trypsin for 4 h. Peptide digests in supernatant were quenched with trifluoroacetic acid (TFA) and finally, desalted with C18 MicroSpin columns as described in [58]. The dried peptides were reconstituted in Buffer A, diluted 1:20 HPLC water containing formic acid and loaded onto Evotips (Evosec, Hamburg, Germany) by manufacturer’s instructions. LC-MS analysis was performed by using the Evosep One liquid chromatography system coupled to a hybrid trapped ion mobility quadrupole TOF mass spectrometer (Bruker timsTOF Pro) via a CaptiveSpray nano-electrospray ion source. Separation was done in 8 cm × 150 µm column with 1.5 µm C18 beads with 60 samples per day methods (21 min gradient time). Mobile phases A and B were 0.1% formic acid in water and 0.1% formic acid in acetonitrile, respectively. The MS analysis was performed in the positive-ion mode using data-dependent acquisition (DDA) in PASEF [59] mode with DDA-PASEF-short_gradient_0.5s-cycletime -method.

Raw data were processed with FragPipe v17.1 utilizing MSFragger [60] against reviewed human entries of the UniProtKB database (downloaded 8.3.2022). N-terminal acetylation and oxidation of methionine were used as dynamic modifications. Biotinylation of lysine and N-termini were set as variable modifications. Trypsin was selected as an enzyme, and a maximum of two missed cleavages were allowed. Both instrument and label-free quantification parameters were left to default settings. Results from these steps are Spectral Counts (SPC) values from peptides with FDR < 0.01 from Philosopher [61]. STRING database was used to create physical networks and retrieve confidence of interaction scores. Illustrations were prepared with R-studio version 2023.06.1 and R v4.3.1 with ggplot2, Cytoscape (v. 3.10.2) and Adobe Illustrator (v. 28.0).

### Analysis of GR and SUMO2/3 RIME MS data

The analysis of quantitative protein group data generated by MaxQuant software, formatted in tables, was performed using a Python pipeline. Data were filtered to exclude contaminants, keratins, mitochondrial, and ribosomal proteins. Further, a filtration step based on SPCs was employed, selecting proteins detected in at least two out of three replicates (SPC > 1) for the protein to be considered expressed and taken for subsequent analysis. For normalization, intensity values were divided by the sum of the total intensity for each sample separately. Additionally, proteins had to pass log_2_[FC] filter against corresponding IgG sample (log_2_[FC] ≥ 2). Proteins were considered Dex-dependent if log_2_[FC] against IgG sample (GR Dex vs IgG Dex) was ≥ 2 and log_2_[FC] against control treatment (GR Dex vs GR DMSO-EtOH) was ≥ 0.5. Normalized intensity values in DMSO for SUMO2/3-associated proteins were used as a proxy for protein SUMOylation level.

### Statistical analysis

Statistical significance was determined with GraphPad Prism software unless otherwise stated using the appropriate tests specified above and in each figure legend. The data meet assumptions of population distribution. Variance between the groups that are being statistically compared is similar. Differences were considered significant at *p* < 0.05. Data are presented as mean ± standard deviation. Statistical significance is indicated in the figures as follows: * = *p* < 0.05, ** = *p* < 0.01, *** = *p* < 0.001.

### Public datasets

Publicly available ChIP-seq datasets were analyzed as previously indicated [17, 55]; GSE67046 for GR ChIP-seq, GSE115494 for H3K27ac ChIP-seq, GSE109377 for H3K4me1 ChIP-seq.

## Supplementary information

Supplementary Figures

Supplementary Table ST1

Supplementary Table ST2

Supplementary Table ST3

Supplementary Table ST4

Supplementary Table ST5

## Data Availability

The MS proteomics data have been deposited to the ProteomeXchange Consortium via the PRIDE [62] partner repository with the following dataset identifier: PXD051304. ChIP-seq, ATAC-seq and RNA-seq datasets have been submitted to the Gene Expression Omnibus database with the accession code GSE233553.
