# Supplementary material for: SUMOylation inhibition potentiates the glucocorticoid receptor to program growth arrest of acute lymphoblastic leukemia cells
Source: Oncogene. 2025 Feb 14;44(18):1259–71. doi: 10.1038/s41388-025-03305-3 (PMC12048349; doi:10.1038/s41388-025-03305-3)
Supplement: Supplementary file 1 — Supplementary Figures [file 41388_2025_3305_MOESM1_ESM.pdf]

Supplementary figures
for

SUMOylation inhibition potentiates the glucocorticoid receptor to program growth arrest of acute lymphoblastic leukemia cells

Emma Valima, Vera Varis, Kseniia Bureiko, Joanna K. Lempiäinen, Anna-Mari Schroderus, Laura Oksa, Olli Lohi, Tuure Kinnunen, Markku Varjosalo, Einari A. Niskanen, Ville Paakinaho, Jorma J. Palvimo

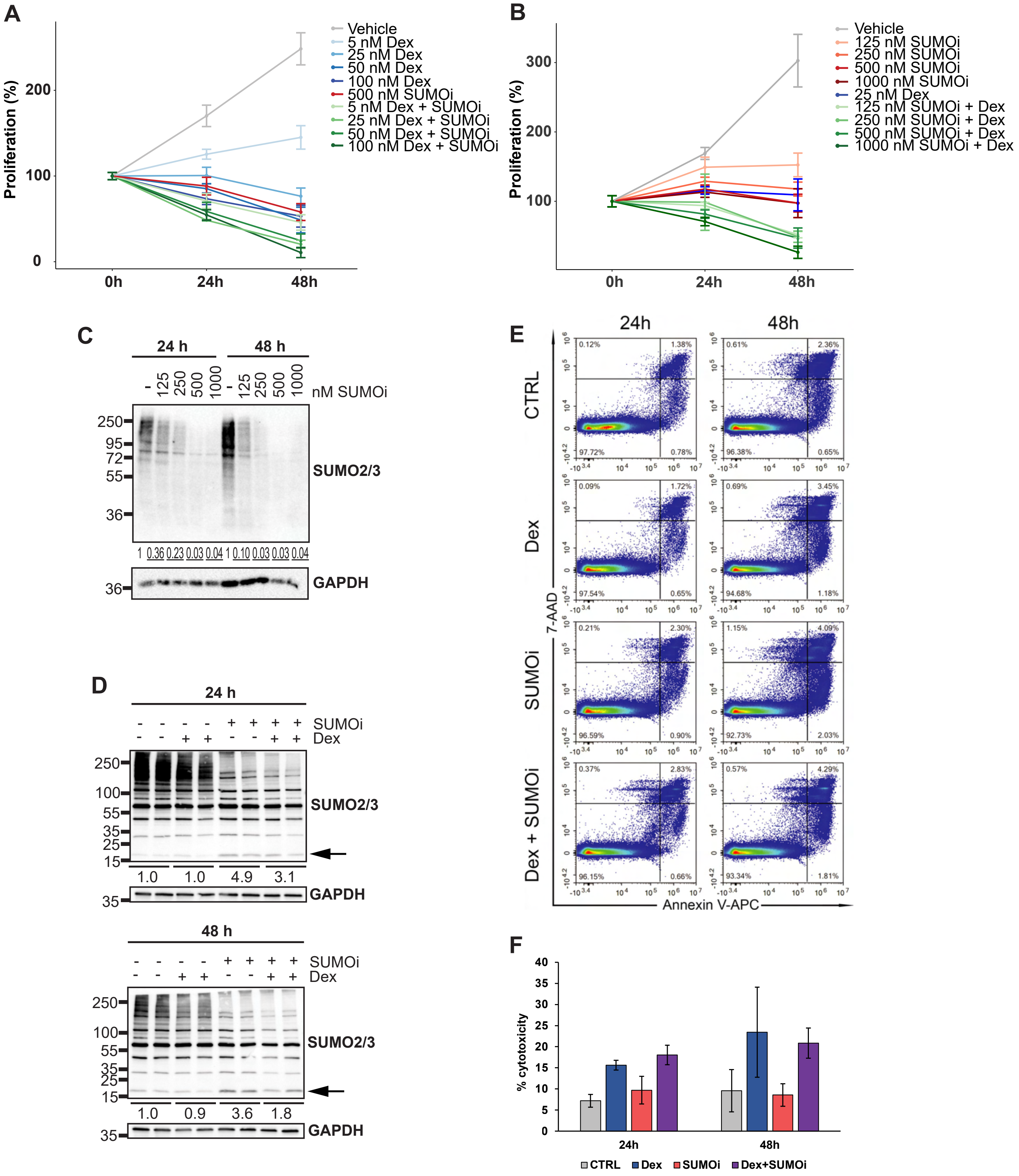

Supplementary Figure S1: (A) Line graph of MTS analysis of NALM6 cells treated with increasing concentrations of Dex and 500 nM SUMOi or combination. (B) Line graph of MTS analysis of NALM6 cells treated with increasing concentrations of SUMOi and 25 nM Dex or combination. (C) Immunoblotting of SUMO2/3 (MBL antibody M114-3) and loading control (GAPDH) in NALM6 cells exposed to increasing concentrations of SUMOi. Numbers below blot indicate normalised GR signal compared to time point vehicle control. (D) Immunoblotting of SUMO2/3 (Zymed antibody 51-9100) showing free, unconjugated SUMO2/3 (arrows) and loading control (GAPDH) in NALM6 cells exposed to vehicle control (DMSO-EtOH), 10 nM Dex, 125 nM SUMOi or combination. Average signal for free SUMO2/3 from two replicates normalized to GAPDH and further to time point vehicle control is indicated below the samples. (E) Representative flow cytometry gating strategy for the apoptosis analysis. (F) Bar graph of cytotoxicity assay results. Bars represent mean of 5 replicates with SD.

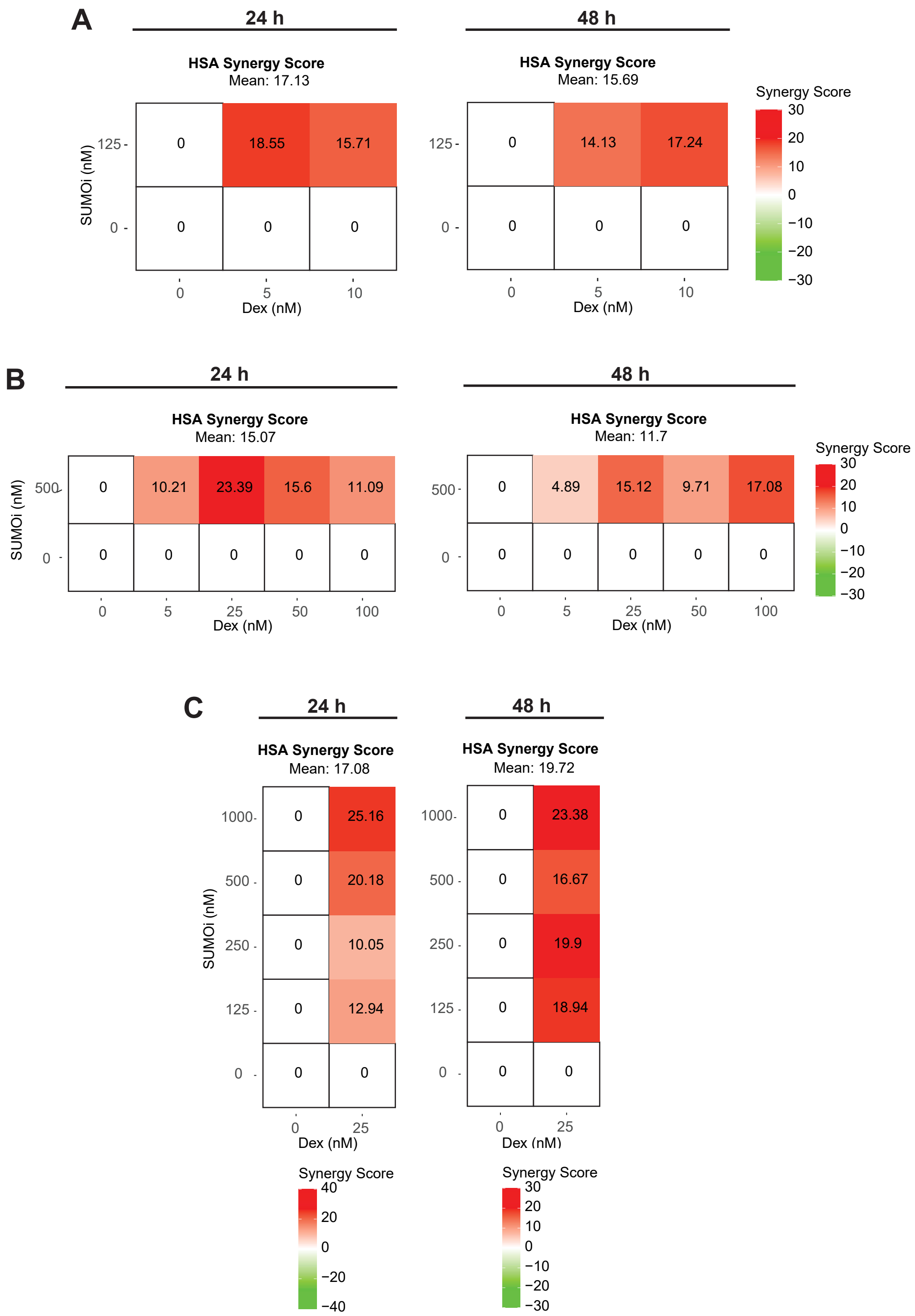

Supplementary Figure S2: Synergy scoring of NALM6 cell MTS assay results. (A) Synergy scores for 5 or 10 nM Dex and 125 nM SUMOi after 24 h and 48 h of exposure were calculated based on HSA reference model (Berenbaum, 1989, Pharmacol Rev). The MTS assay results that were used for calculating the scores are presented in Figure 1A. (B) Synergy scores for 500 nM SUMOi with variable concentrations of Dex after 24 h and 48 h of exposure were calculated based on HSA reference model. The MTS assay results that were used for calculating the scores are presented in Supplemental Figure S1A. (C) Synergy scores for 25 nM Dex with variable concentrations of SUMOi after 24 h and 48 h of exposure were calculated based on HSA reference model. The MTS assay results that were used for calculating the scores are presented in Supplemental Figure S1B.

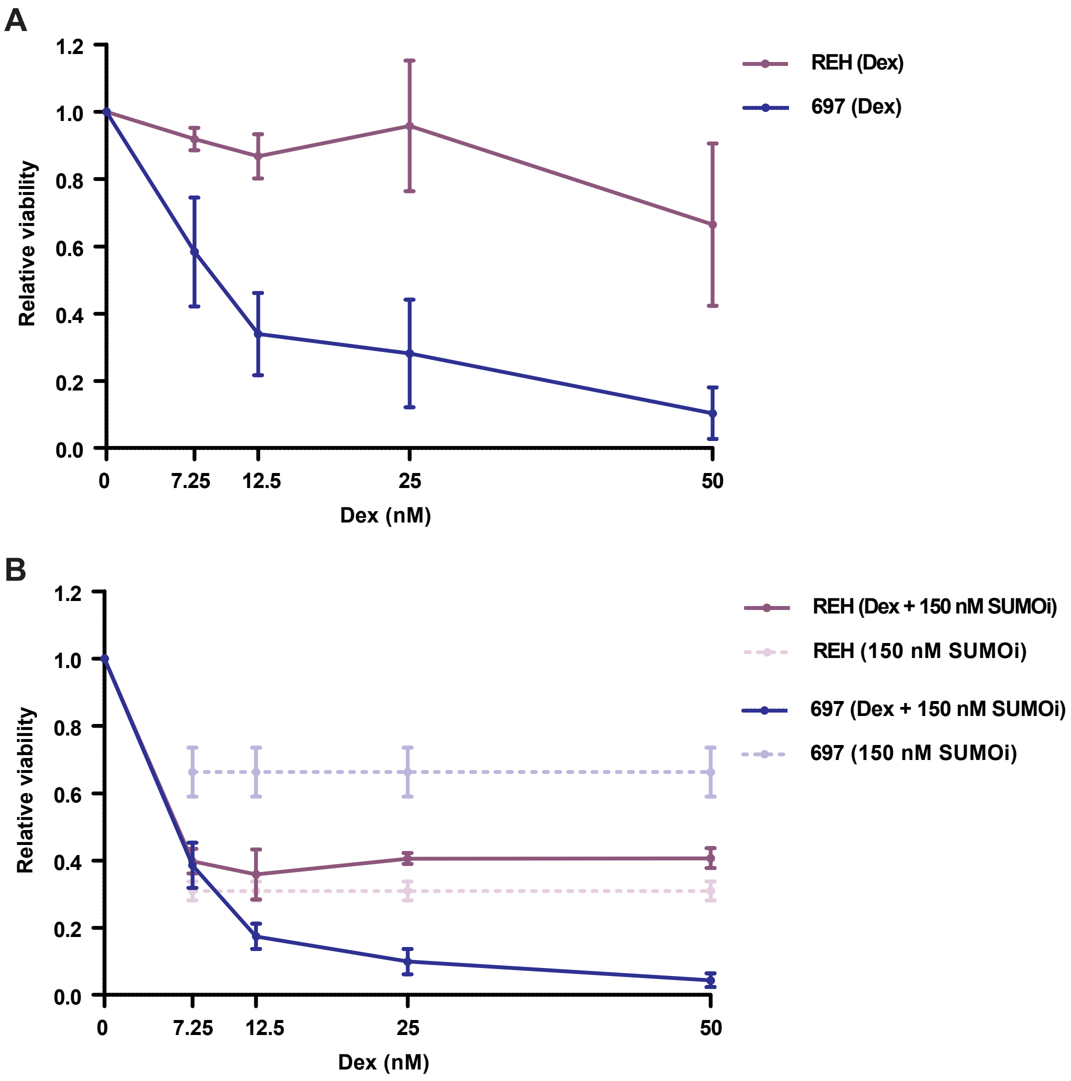

C

B-ALL cell line	Genetic subtype	GC responsiveness
NALM6	DUX4-IGH fusion, ERG deletion	GC sensitive
697	TCF3-PBX1 (E2A-PBX1) fusion	GC sensitive
REH	ETV6-RUNX1 fusion, NR3C1 deletion	GC resistant

Supplementary Figure S3: Viability of REH and 697 cells exposed to Dex, SUMOi or combination. REH or 697 cells plated into 96-well plates (10 000 cells/well) were treated with SUMOi (150 nM) only, Dex only (0-50 nM), and with their combination where SUMOi concentration was constant (150 nM), and Dex concentration varied (0-50 nM). After incubation for 72 h, cell viability was measured using AlamarBlue assay (Invitrogen, Carlsbad, CA, USA) according to manufacturer's instructions. The results were normalized using DMSO-treated cells to obtain relative viability. (A) The effect of Dex on REH and 697 cell viability. (B) The effect of Dex+SUMOi combination on REH and 697 cell viability. Data points represent mean of three biological replicates with SD. (C) Table of B-ALL cell lines used, listing major genetic translocations and deletions, and responsiveness to glucocorticoids (GCs).

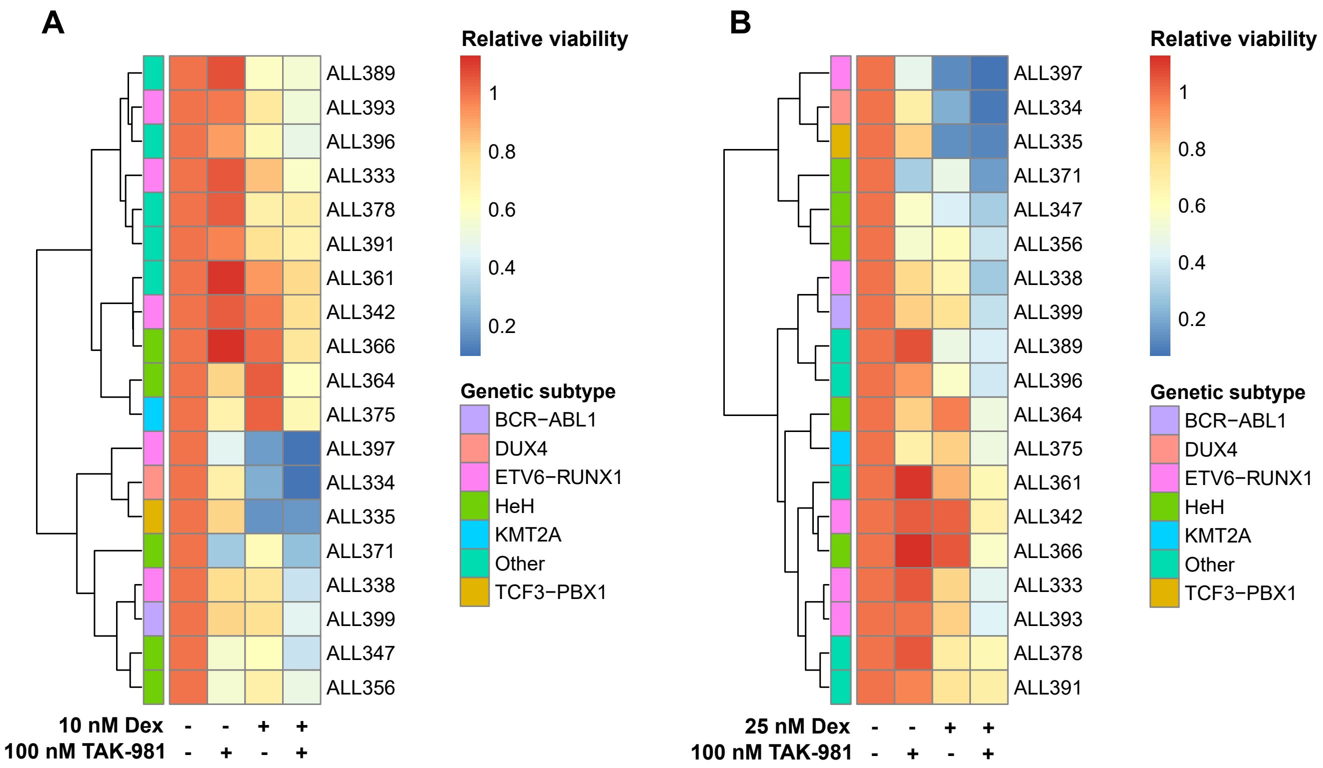

Supplementary Figure S4: Heatmaps showing relative viability of cells isolated from 19 B-ALL patients after treatment with vehicle control, 10 nM Dex (A), 25 nM Dex (B), 100 nM TAK-981 or Dex+TAK-981 combination for 48 h *ex vivo*. HeH, high hyperdiploid karyotype.

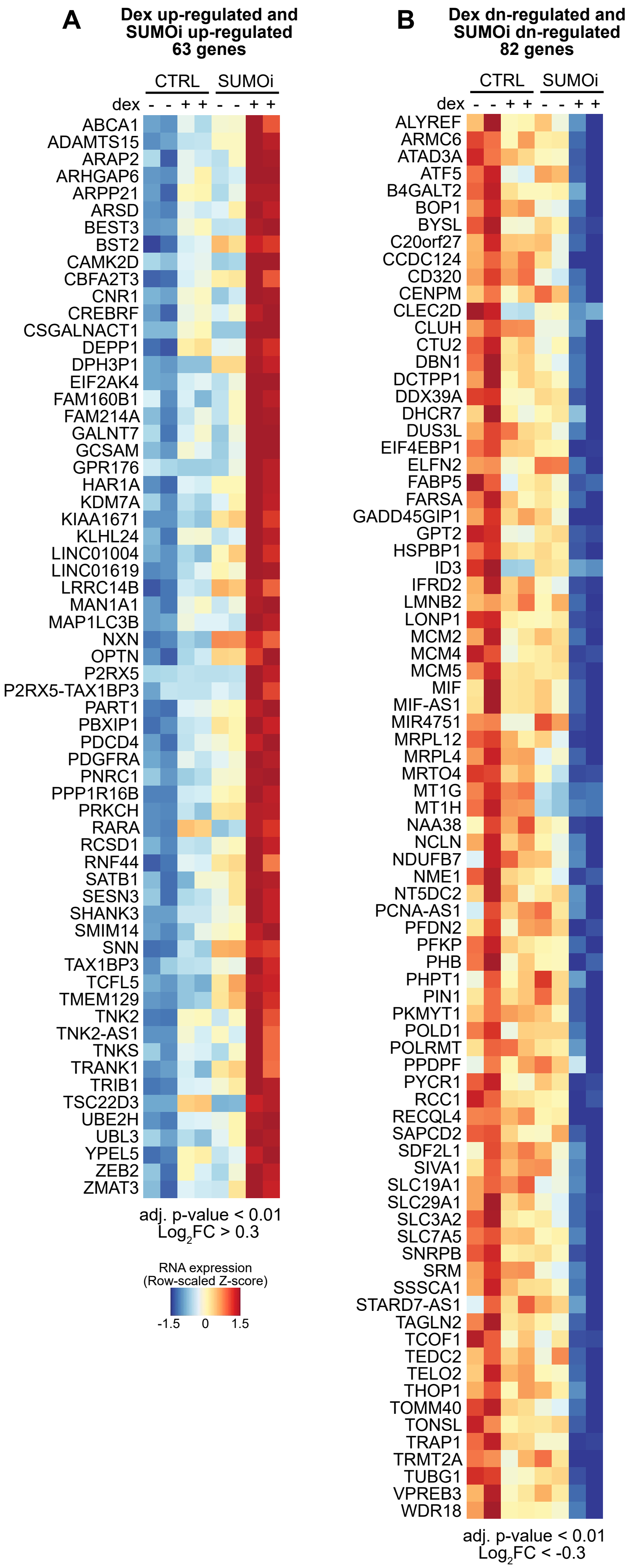

Supplementary Figure S5: Heatmaps containing all genes in Dex-up/SUMOi-up (A) and Dex-dn/SUMOi-dn (B) clusters.

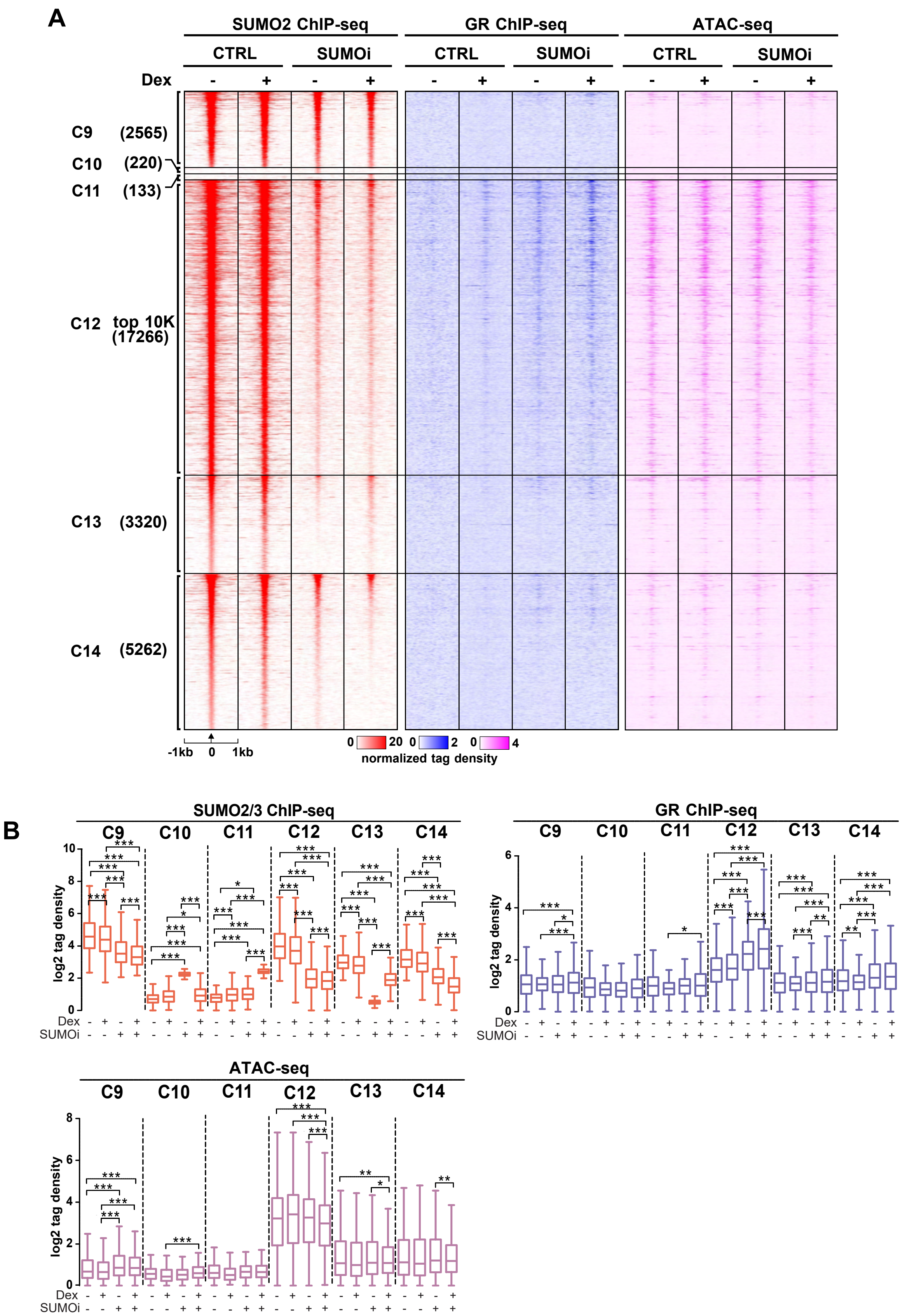

Supplementary Figure S6: (A) Heatmap of SUMO2/3 and GR ChIP-seq and ATAC-seq signals in SUMO2/3 binding sites. C9 = sites with moderate differences in SUMO2/3 occupancy between SUMOi and Dex+SUMOi treated cells, C10 = sites with increased SUMO2/3 occupancy upon SUMOi exposure, C11 = sites with increased SUMO2/3 occupancy upon Dex+SUMOi exposure, C12 = sites with decreased SUMO2/3 occupancy in response to both SUMOi alone and the combination of Dex and SUMOi, C13 = sites with strongly decreased SUMO2/3 occupancy in response to SUMOi, C14 = sites with greater decrease in SUMO2/3 occupancy upon Dex+SUMOi treatment. (B) Boxplots of SUMO2/3 and GR ChIP-seq and ATAC-seq signal (tag density) in clusters depicted in (A). Statistical significance was calculated with One-way ANOVA with Bonferroni post hoc test, with asterisks denoting statistical significance: * = $p < 0.05$, ** = $p < 0.01$, *** = $p < 0.001$.

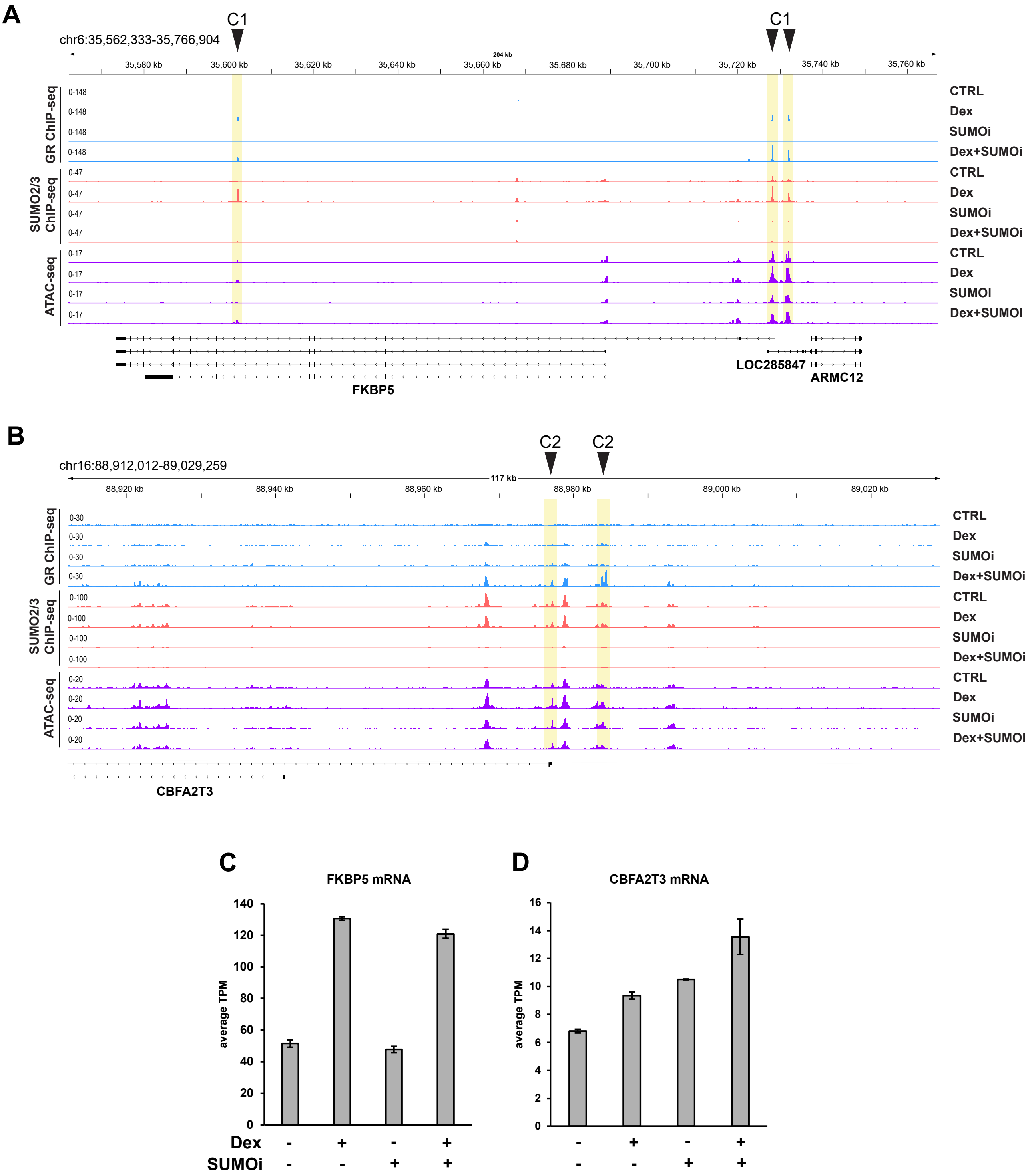

Supplementary Figure S7: (A) Example IGV track showing C1 GRBs (arrows) localised within or near FKBP5 locus. (B) Example IGV track showing C2 GRBs (arrows) localised within or near CBFA2T3 locus. (C) and (D) Transcripts per million (TPM) counts for FKBP5 (C) and CBFA2T3 (D). Bars represent average TPM of two replicates with SD.

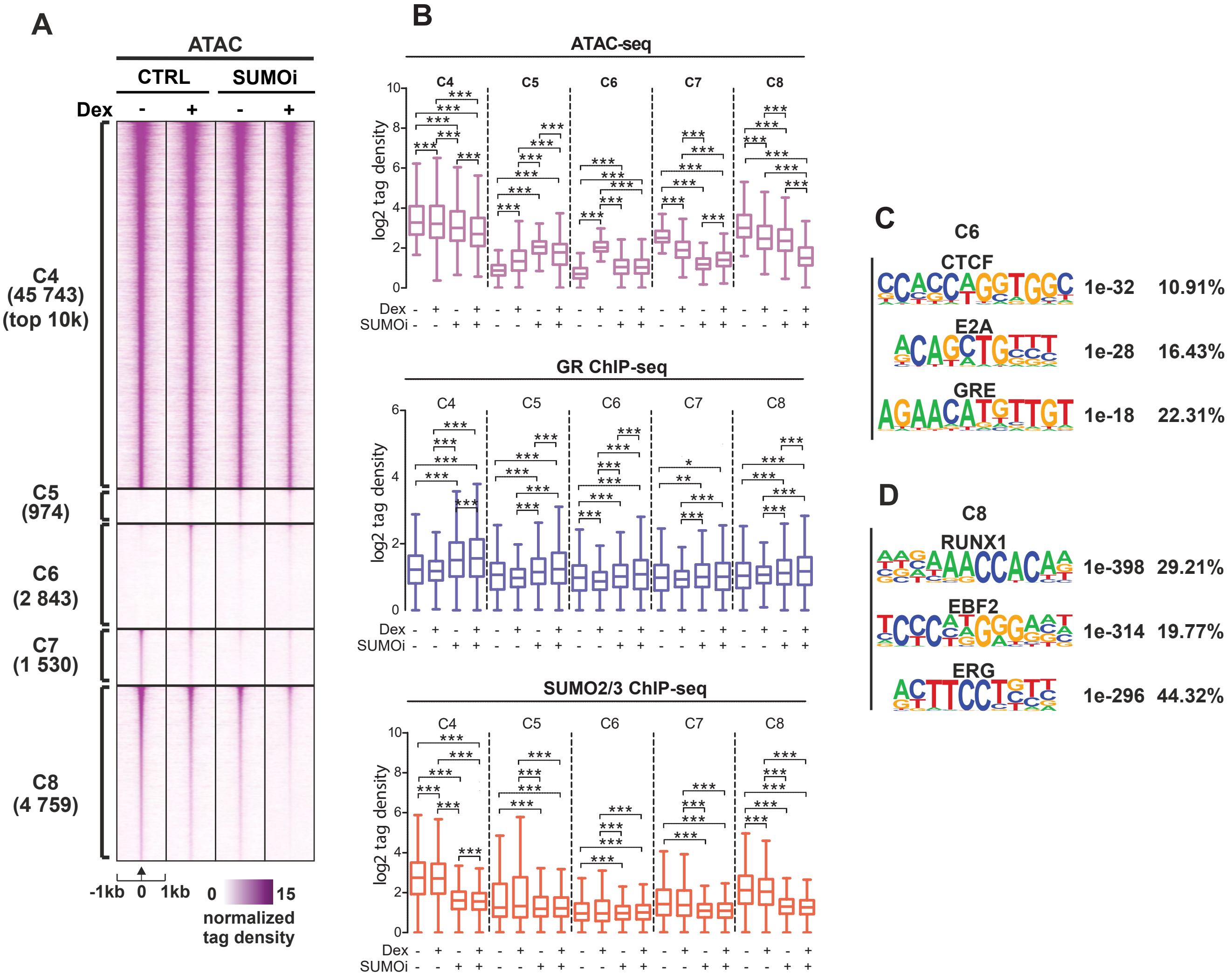

Supplementary Figure S8: (A) Heatmap of ATAC-seq signal. (B) Boxplots of ATAC-seq, GR ChIP-seq and SUMO2/3 ChIP-seq signal in C4-C8 clusters (tag density). (C) Top three de novo motifs for C6. (E) Top three de novo motifs for C8. Statistical significance for boxplots was calculated with One-way ANOVA with Bonferroni post hoc test. Asterisks denote statistical significance: * = p < 0.05, ** = p < 0.01, *** = p < 0.001.

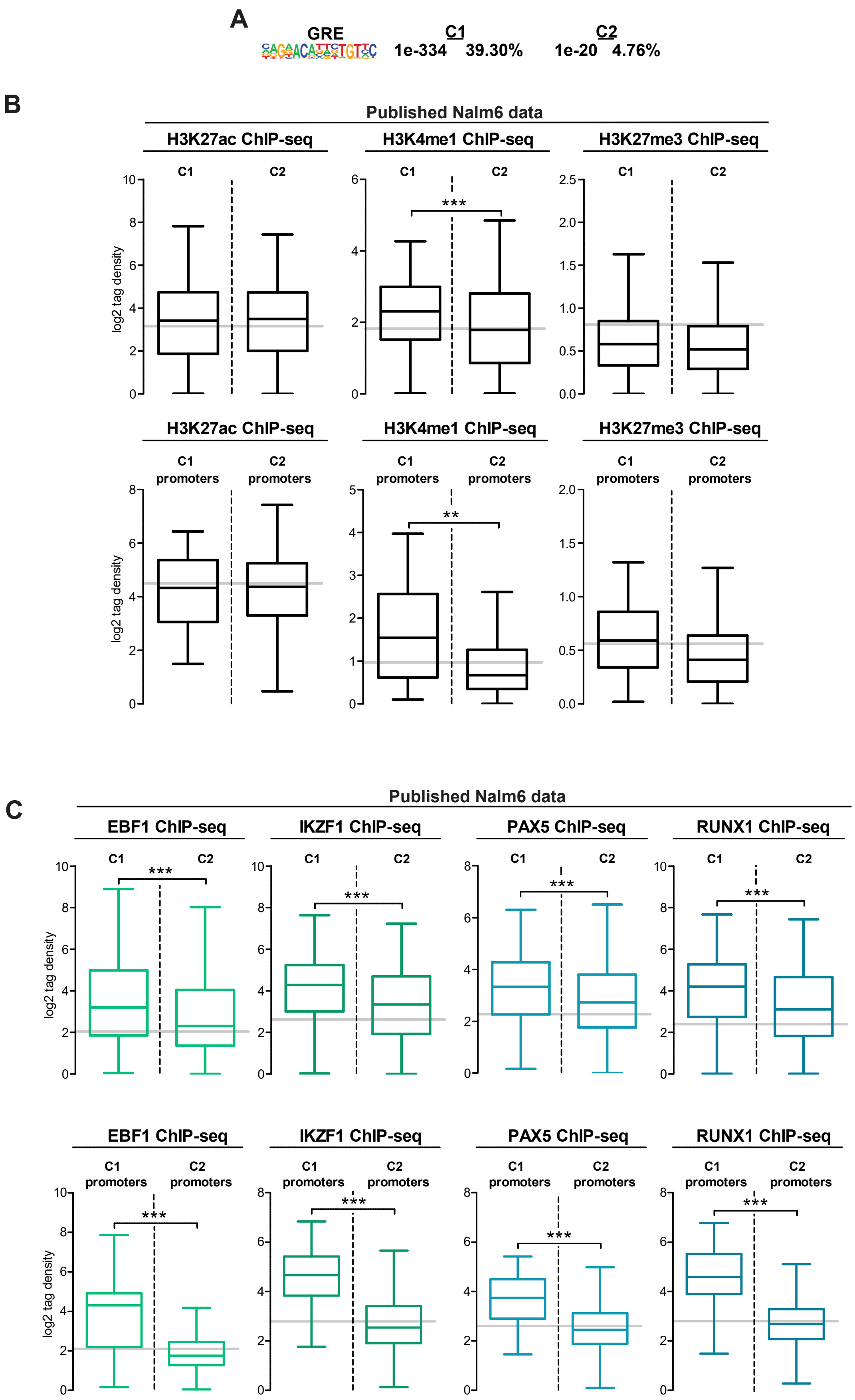

Supplementary Figure S9: (A) Enrichment of known GRE motif in C1 and C2 GRB clusters, showing p-values and percentage of target sites with motif. (B) Boxplots of published ChIP-seq data for indicated histone markers in whole C1 and C2 populations (top panel) and promoter subpopulations (bottom panel). (C) Boxplots of published ChIP-seq data for indicated TFs in whole C1 and C2 populations (top panel) and promoter subpopulations (bottom panel). Statistical significance was calculated with Two-tailed t-test. Asterisks denote statistical significance: * = p < 0.05, ** = p < 0.01, *** = p < 0.001. Grey lines indicate the average $\log_2$ tag density in pooled input samples.

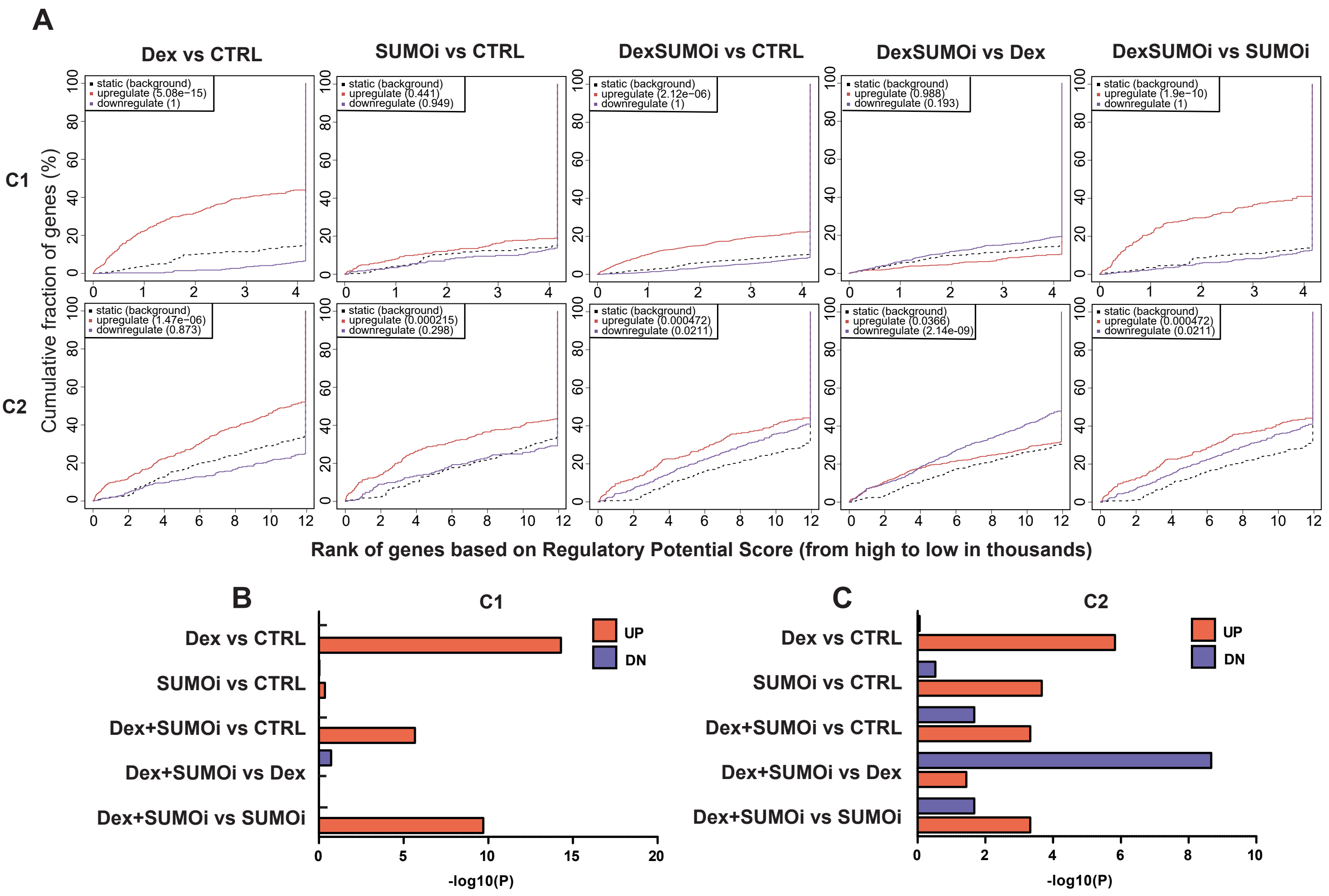

Supplementary Figure S10: (A) Functional prediction matrices from BETA analysis for GRBs in C1 and C2 within each treatment comparison. Background of non-regulated genes is indicated with black dashed line, cumulative GR activation function with red line and cumulative GR repression function with purple line. Genes are cumulated by rank based on the regulatory potential score, from high to low. P-values from the Kolmogorov-Smirnov test for difference compared to background are indicated in the top left corner of each plot, with a p-value < 0.05 considered as significant. (B) and (C) Bar graphs depicting -log10 transformations of p-values from (A) for C1 and C2, respectively.

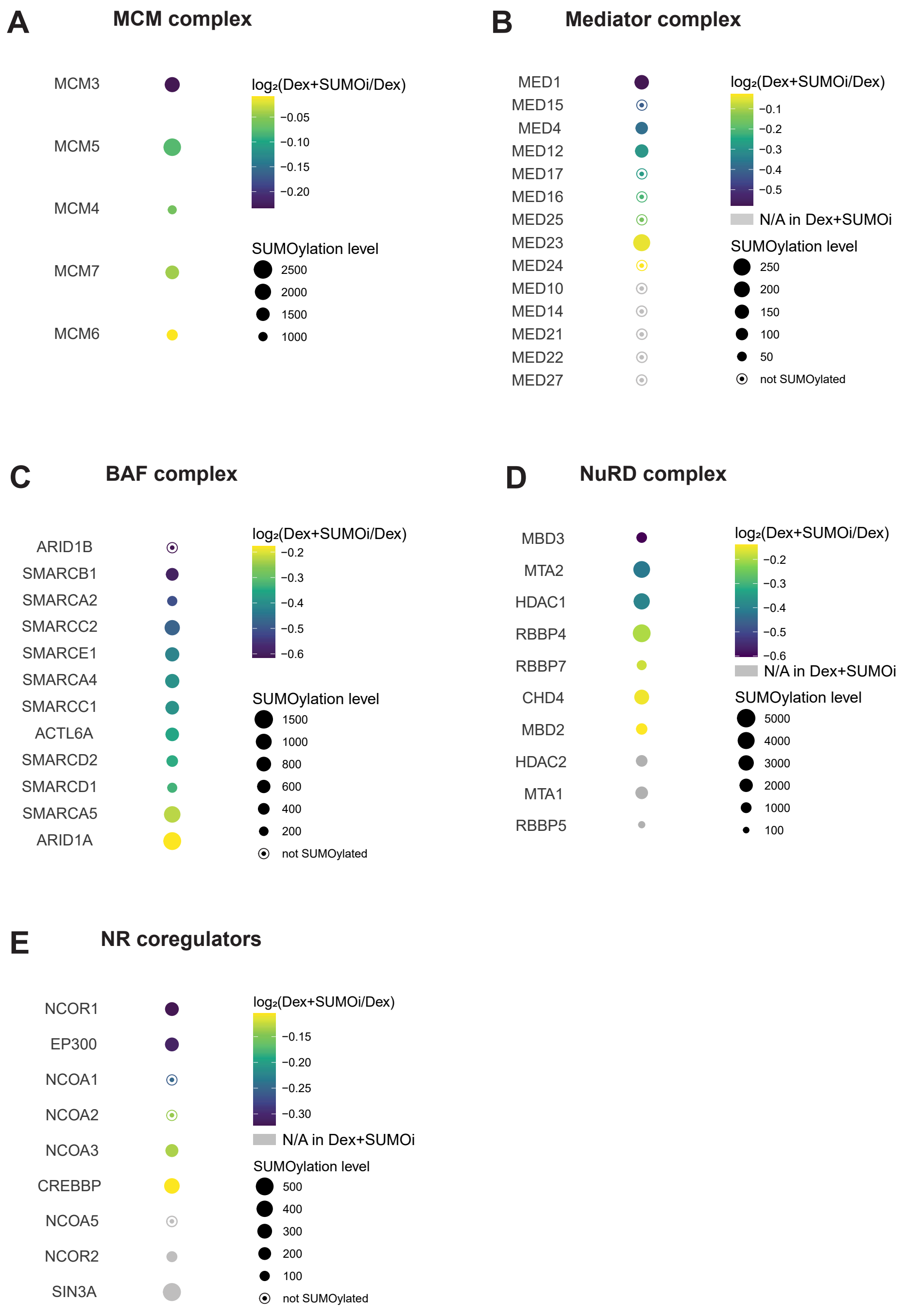

Supplementary Figure S11: Dot plots showing SUMOi-effect (log_2[Dex+SUMOi/Dex]) on coregulators and other selected proteins identified as part of Dex-dependent GR chromatome. Size of dot corresponds to SUMOylation level of the protein by showing average normalized intensity in DMSO (SUMO2/3). (A) Identified MCM complex members, (B) identified mediator complex members, (C), identified BAF complex members, (D) identified NuRD complex members, (E) identified nuclear receptor (NR) coregulators.

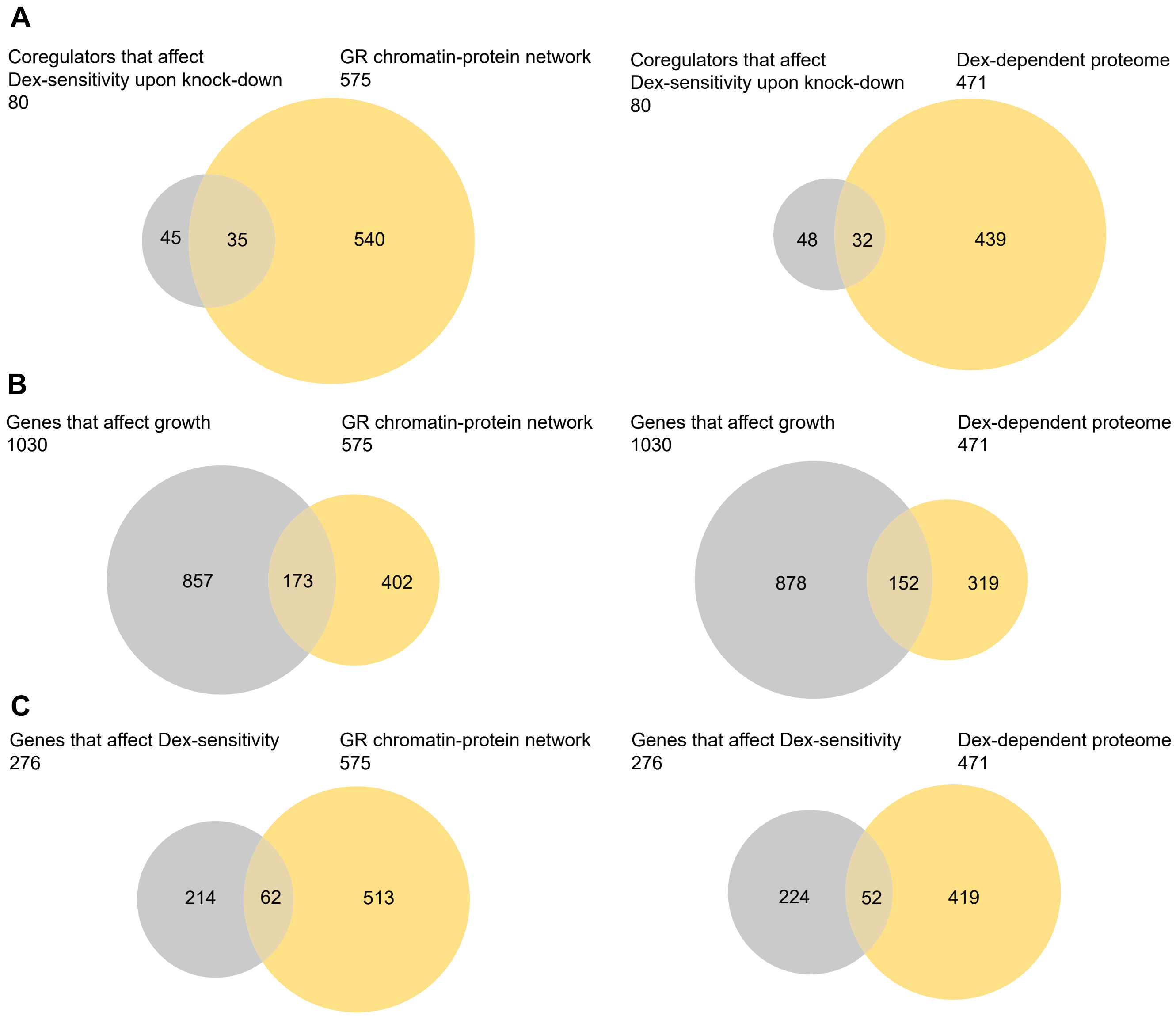

Supplementary Figure S12: (A) Venn diagrams showing the overlap of a published dataset of coregulators (ref. 28) that affect Dex-sensitivity upon knock-down and the whole identified GR chromatome (left panel) or Dex-dependent chromatome only (right panel). (B) Venn diagrams showing the overlap of a published dataset of genes that affect growth in NALM6 cells and the whole identified GR chromatome (left panel) or Dex-dependent chromatome only (right panel). (A) Venn diagrams showing the overlap of a published dataset of genes that affect Dex-sensitivity in NALM6 cells and the whole identified GR chromatome (left panel) or Dex-dependent chromatome only (right panel).
